# Mechanism-Based and Experimentally Calibrated Modelling Strategy for Prestressed I-Shaped UHPFRC–Steel Composite Beams with Continuous Shear Connectors

**DOI:** 10.3390/ma19214644

**Published:** 2026-10-31

**Authors:** Peter Harsányi, Norbert Randl, Nguyen Viet Tue

**Affiliations:** 1 Faculty of Civil Engineering and Architecture, Carinthia University of Applied Sciences (CUAS), Villacher Straße 1, A-9800 Spittal, Austria; n.randl@cuas.at; 2 Institute of Structural Concrete, Graz University of Technology, Lessingstraße 25, A-8010 Graz, Austria; tue@tugraz.at

**Keywords:** finite element modelling, ATENA Science, UHPFRC, continuous shear connector, composite beam, digital image correlation, prestressing

## Abstract

This paper presents a finite element investigation of prestressed I-shaped Ultra-High-Performance Fibre-Reinforced Concrete (UHPFRC)–steel composite beams with continuous puzzle-shaped shear connectors. Experimentally derived material and bond relationships are implemented in three-dimensional models in ATENA Science. The tensile response is identified from flexural tests evaluated by digital image correlation and specimen-level inverse analysis, while the compression input is derived from cube, cylinder and prism tests. Beam I-2 is used for structural model selection. Separate studies assess the tensile reference-size sensitivity at a fixed mesh and the mesh sensitivity at a fixed reference size. The selected 35 mm mesh and 25 mm Tension Characteristic Size are subsequently transferred to I-1 and I-3 without further specimen-specific adjustment. For I-1 to I-3, peak-load deviations are +10.1%, +4.9% and +0.7%, respectively; the corresponding deflection deviations are +8.3%, +1.0% and −12.7%. The models reproduce strand-controlled ultimate failure and qualitatively reproduce the observed steel-web strain-localisation patterns. An additional I-4 analysis reaches strand rupture instead of the experimentally observed tensile-flange splitting and overestimates its peak load by 25.4%, demonstrating a boundary of validity of the adopted symmetry-constrained model. Internal tensile splitting demand, compressive inelasticity and transverse flange deformation are interpreted numerically within these limitations.

## 1. Introduction

Ultra-high performance fibre-reinforced concrete (UHPFRC) is increasingly used in composite structures for its very high compressive strength, significant post-cracking tensile capacity and excellent durability [1,2,3,4,5]. When combined with a high-strength S460 ML steel girder and prestressing strands in the tensile zone, this combination enables thin flanges and slender cross-sections with high load-bearing and deformation capacities. In recent years, several concepts for UHPFRC–steel composite members have been proposed, ranging from ultra-high-performance concrete (UHPC) overlays on existing bridges to newly designed conventional UHPC and composite girders with UHPC slabs and various types of mechanical shear connectors [6,7,8,9].

Conventional applications rely on headed studs to connect the steel web and concrete compression flange. In high-strength concrete, these connectors often cannot meet the ductility criterion in Eurocode 4, EN 1994-1-1, Annex B.2.5.(4) [10], for a minimum of 6 mm after a 10% drop in maximum load. The high resistance of the surrounding UHPFRC can cause stud shear failure even with increased steel cross-sections [11,12,13,14,15,16]. To fully utilise the mechanical properties of UHPFRC and steel, continuous shear connectors have been proposed as an alternative [17,18]. Their behaviour has been investigated experimentally and theoretically: full-scale push-out tests identified the influence of steel thickness, steel grade and dowel geometry on the shear resistance and demonstrated that plastic deformation of the steel dowel governs the subsequent concrete failure [19,20], while mechanically based models for the pry-out resistance and for the nonlinear shear–slip characteristic have been derived and consolidated into design provisions [21,22]. These approaches were developed primarily for normal- and high-strength concrete, whereas their extension to UHPFRC, where matrix strength, fibre bridging and confinement alter both the resistance and the failure kinematics, remains to be further investigated. Such connectors provide a more uniform shear flow, reduce local stress peaks and allow for a more ductile failure mechanism. A preliminary test campaign investigated the failure modes of such continuous shear dowels embedded in UHPFRC with high-strength prototype steel fibres, using digital image correlation (DIC) [23]. The design of those dowels was based on [24]. The tests covered different connector geometries, reinforcement layouts and UHPFRC fibre contents and characterised the associated failure modes.

A subsequent large-scale series tested four prestressed I-shaped composite girders with UHPFRC flanges connected by puzzle-shaped continuous connectors in four- and three-point bending, with different internal reinforcement layouts [25]. The beams were prestressed by bonded seven-wire strands located in the tensile flange. Beams I-1 to I-3 reached high capacities and strand-controlled failure, whereas I-4 failed by longitudinal splitting. The conducted research demonstrated the structural efficiency of the proposed design but also raised questions that cannot be answered with conventional measurement systems or sensors alone. In particular, the simultaneous interaction of strain-hardening tensile behaviour, confinement-sensitive compression and bond-controlled prestress transfer creates a multi-mechanism stress state that is not experimentally accessible in a spatially resolved manner. The internal stress redistribution between UHPFRC, high-strength S460 ML steel web, puzzle-shaped continuous connectors and the prestressing strands in the plastic range of material behaviour could not be directly observed. As a consequence, a modelling approach capable of consistently linking material-level behaviour to structural response is required.

Finite element (FE) modelling can address these questions if its material models reproduce the complex behaviour of UHPFRC under tensile and compressive loading and the bond behaviour of prestressing strands and reinforcement. For conventional and high-strength concrete, numerous constitutive laws and modelling approaches are available and have been validated extensively [26,27,28,29]. UHPFRC behaviour is strongly affected by fibre type, orientation and volume fraction, mix design, curing conditions and confinement [30,31,32]. In prestressed UHPFRC–steel composite members, these influences interact with bond-controlled prestress transfer and connector-induced confinement, such that modelling accuracy is governed by the consistency of the material laws across multiple stress states. A reliable FE simulation therefore requires careful calibration of the tensile and compressive stress–strain relationships and of the bond laws based on dedicated material and bond tests for the specific UHPFRC used.

Recent numerical studies on steel–UHPC composite members reproduce global behaviour at different levels of detail, while differing in their treatment of local mechanisms and constitutive response. Chen et al. [33] developed three-dimensional ABAQUS models of UHPC-encased steel composite beams and validated both the global load–deflection response and the experimentally observed damage patterns against tests supported by digital image correlation. At the connector level, Zhao et al. [34] combined pull-out tests of steel dowel connectors in UHPC with three-dimensional concrete damaged plasticity (CDP) analyses to reproduce load–slip and load–strain responses and to investigate the influence of connector geometry. At a reduced modelling scale, Xiao et al. [35] proposed a computationally efficient fibre beam-column formulation combined with a smeared-crack approach. The method reproduced load–deflection curves, ultimate capacities and crack-width development of a broad range of steel–UHPC members with only a fraction of the computational effort required by detailed three-dimensional models, but inherently does not resolve three-dimensional stress states or local load-transfer mechanisms around individual connectors. Closest to the present structural configuration, Matanmi et al. [36] recently tested and numerically analysed a full-scale high-strength steel–UHPC composite beam with modified clothoid-shaped dowels. Their modelling strategy combined a global beam model with a detailed solid model for the connector regions and was subsequently used for parametric studies on UHPC slab and steel-flange thickness.

A further distinction concerns how the nonlinear UHPC material response is defined. In the detailed model of Chen et al. [33], material-specific tensile strength, elastic modulus and strain-hardening parameters were obtained from accompanying cube and direct dog-bone tensile tests. However, the complete constitutive response was not derived directly from these tests: the compressive stress–strain relationship followed an existing analytical formulation, the tensile response was represented by a previously proposed strain-hardening model, and the tensile and compressive damage evolution functions were adopted from separate literature sources. Thus, experimentally determined properties of the investigated UHPC were used to parameterise established constitutive formulations rather than to define all branches of the material law directly. In the connector study of Zhao et al. [34], experimentally determined basic UHPC properties were reported, and a CDP formulation was employed, but the complete constitutive parameter set and the underlying tensile and compressive stress–strain relationships were not documented, limiting the reproducibility of the material model. Matanmi et al. [36] similarly employed the CDP framework, but adopted the compressive constitutive relationship from other sources; characteristic strains governing the curve shapes were prescribed according to these formulations rather than obtained from dedicated post-peak tests on the UHPC of the investigated beam. The reduced-order approach of Xiao et al. [35] uses analytically parametrised uniaxial relationships: its proposed compressive law requires compressive strength, elastic modulus and peak strain, for which empirical estimation equations are provided if test data are unavailable, while its tensile formulation requires only tensile strength and elastic modulus from testing and derives the remaining characteristic parameters empirically.

These approaches demonstrate that analytically parametrised constitutive laws can reproduce global structural behaviour. However, modelling prestressed I-shaped UHPFRC–steel members with UHPFRC in both the tensile and compressive flanges requires a consistent representation of tensile strain hardening, compression softening, bond-controlled prestress transfer and connector-induced confinement throughout the highly ductile response up to the ultimate limit state (ULS). To the authors’ knowledge, a modelling strategy combining experimentally identified nonlinear tensile and compressive branches for this specific prestressed system with continuous puzzle-shaped connectors, and explicitly separating material identification from subsequent member-level model selection and transfer assessment, has not yet been reported. The contribution is a strategy for assessing how far experimentally informed inputs can reproduce this coupled response and reveal internal load-transfer mechanisms without extensive member-specific parameter fitting.

Building on the two experimental campaigns [23,25], this paper develops and assesses a three-dimensional (3D) FE model of a prestressed I-shaped UHPFRC–steel girder with continuous puzzle-shaped shear connectors. The model is implemented in ATENA Science 2025u and GiD 17.0.1 using user-defined material laws for the UHPFRC, the prestressing strands and the S460 ML steel web, based on compressive, tensile and flexural tests, as well as bond tests on prestressing strands. Particular attention is paid to (a) the translation of flexural tension tests into an equivalent uniaxial tensile stress–strain relationship using a characteristic crack-band length, (b) the correction of compressive stress–strain curves for testing-machine and specimen effects, and (c) the modelling of bond–slip behaviour of the prestressing strands based on pull-out tests and anchorage-zone measurements in comparison with available research data. Where direct experimental identification was not possible, independently available material data, manufacturer information or mechanically justified modelling assumptions were documented separately. Inverse identification of the material-curve shapes was performed at the material-specimen scale. Subsequent member-level selection of the compression-law alternative, mesh and tensile material-reference size used I-2 and is distinguished from the transfer assessment on I-1 and I-3.

The material models are implemented in a detailed 3D FE model representing the steel web, UHPFRC flanges, continuous connectors and bonded prestressing strands. A longitudinal symmetry plane reduces the computational effort. I-2 serves as the structural reference case for model selection; the selected settings are then transferred to I-1 and I-3 without further adjustment. Assessment covers the global load–deflection response, the strand-controlled ultimate failure and a qualitative comparison with DIC strain-localisation patterns. I-4 is additionally simulated to assess the model boundary for longitudinal splitting. Internal FE fields are used to interpret stress redistribution and connector mechanisms, without treating these internal states as directly measured or independently validated quantities.

The specific objectives are to: (i) establish the tensile, compressive and bond inputs from the available material tests and documented assumptions; (ii) assess constitutive and numerical choices on I-2 and transfer the selected settings to I-1 and I-3; (iii) examine the limitation for splitting-controlled failure using I-4; and (iv) interpret internal connector mechanisms within the stated evidence limits. The study forms the numerical part of a broader research programme on prestressed UHPFRC–steel composite systems; exhaustive parameter sensitivity analysis is outside its scope.

## 2. Experimental Basis—Overview of Previous Testing Program

The same UHPFRC was used throughout both preceding experimental campaigns and the present numerical study, which ensures material consistency between the calibration tests and the structural validation. The mixture was developed and supplied by a project partner at Graz University of Technology following a stepwise particle-packing optimisation according to [37]; its development was not part of the present work, and the detailed mix proportions have not been released for publication. The exact composition is therefore characterised here by its constituent classes and the parameters governing the mechanical response, while the initial constitutive relationships relevant to the numerical model are derived from the dedicated material tests reported in Section 3.1.4. Particular care was taken during casting to achieve a homogeneous fibre distribution by manual placement with short flow paths, in order to avoid fibre segregation in the slender tension flange of the composite beams. Table 1 provides an overview of the used materials and their properties.

The present numerical investigation is based on the findings of the two previously conducted experimental campaigns by the authors. The first campaign focused on the shear transfer mechanism and failure modes of continuous puzzle-shaped steel dowels embedded in UHPFRC with high-strength prototype fibres and conventional reinforcement [23], while the second campaign investigated the global structural behaviour of prestressed I-shaped UHPFRC–steel composite girders, utilising puzzle-shaped connectors to connect the steel web to the compression flange and the prestressed tensile flange [25].

**Figure 1:**
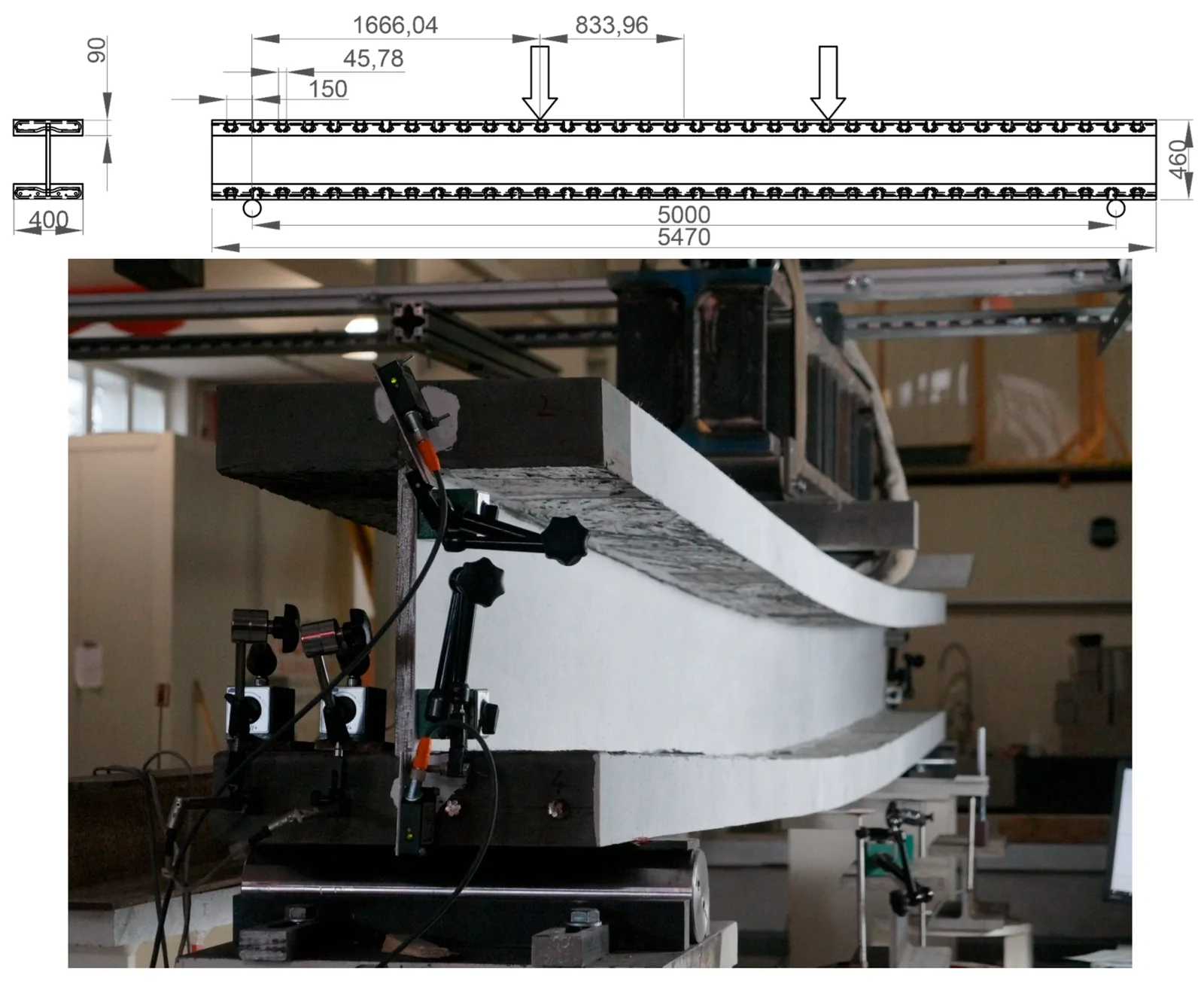
Beam dimensions in mm (**top**) and test layout of experimental campaign (**bottom**).

Four specimens were tested in the experimental campaign. The first girder, I-1, was tested in a three-point bending setup, while beams I-2 to I-4 were tested in a four-point bending setup. The second variation concerned the reinforcement layout. I-1 and I-2 had a complete reinforcement layout, I-3 lacked dowel reinforcement, and I-4 had only prestressing strands without additional conventional reinforcement. Specimens I-1 to I-3 failed at 892.62 kN, 1330.96 kN and 1367.73 kN, respectively, due to rupture of at least one prestressing strand. Beam I-4 failed in the loading phase at 1056.01 kN, due to splitting of the tensile UHPFRC flange along the axis of the puzzle-shaped shear connector. Figure 1 shows the cross-sectional geometry and four-point bending setup.

**Figure 2:**
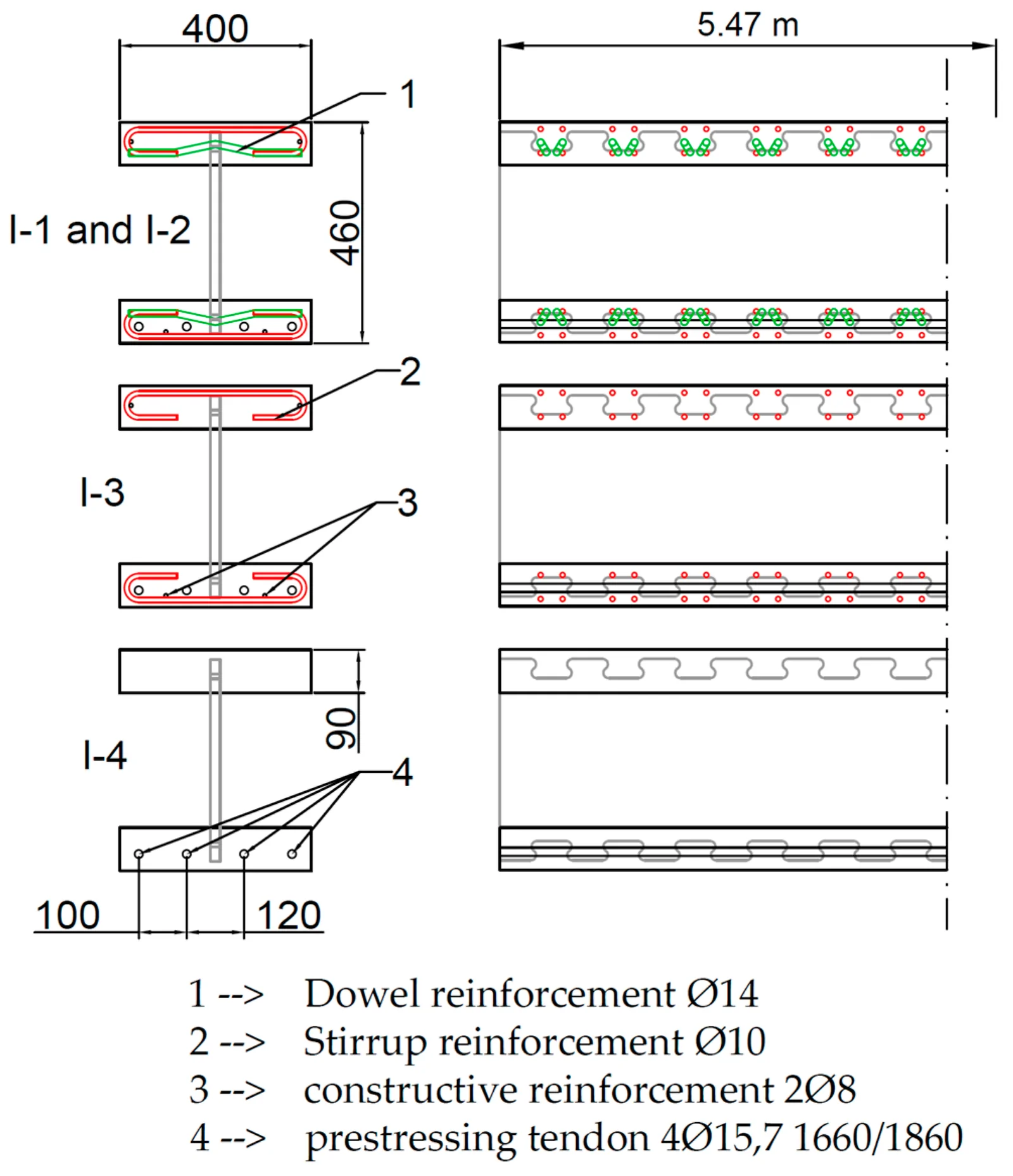
Reinforcement layout and size of the I-shaped beams [mm].

Further details on the reinforcement layout, measurement setup and failure modes can be found in Figure 2.

Beam I-2, which contained the complete conventional reinforcement layout and was tested in four-point bending, was selected as the structural reference for model selection and sensitivity assessment. The selected modelling strategy was subsequently transferred to I-1 and I-3 without further specimen-specific adjustment. I-4, which exhibited tensile-flange splitting rather than strand-controlled failure, was additionally simulated as a boundary-of-validity case. Its numerical response is reported in the global comparison, but it is excluded from the detailed constituent-field assessment of the strand-controlled specimens.

## 3. Finite Element Investigation

The objective of the finite element (FE) analysis of the prestressed UHPFRC–steel composite girders was to address the open questions arising from the previous large-scale experimental campaign. Due to the complex geometry and the combination of high-performance materials, the internal stress states, particularly in the region of the continuous shear connectors, could not be sufficiently quantified in the experiments. The measurement techniques available at the time reached their limits, and post-test sectioning along the connector axis was not feasible without damaging the relevant microstructural details in the UHPFRC, especially in view of the expected partial crushing of the concrete in this region.

The analytical approach adopted for the ultimate limit state in the experimental campaign indicated that the high-performance materials were fully utilised up to failure [25]. The commercial FE software ATENA Science (version 2025u) allows the simulation of UHPFRC and post-peak behaviour through sophisticated constitutive models and user-defined material laws tailored to concrete. Therefore, a numerical investigation was carried out to supplement the experimental observations from both the small-scale tests [23] and the large-scale girder tests [25], and to provide additional insight into the internal stress redistribution and failure mechanisms of the composite system.

### 3.1. Material Properties and Implemented Models

In the following, the fundamental aspects of the selected material models are explained. All input parameters for ATENA Science are provided in the respective appendix. Wherever possible, the material response was derived from dedicated laboratory testing and assessed by separate specimen-level calibration analyses. For the UHPFRC tensile and compressive relationships, representative constitutive models from the literature were additionally used as independent plausibility references but not as calibration targets. Due to the complexity of the numerical modelling task resulting from the combination of several high-performance materials, engineering-based assumptions were required for selected parameters, which could not be fully identified from the experimental campaign on prestressed UHPFRC–steel composite girders.

#### 3.1.1. S460 ML Steel Material Properties

For the material modelling in both experimental campaigns, it was necessary to determine the mechanical properties of the S460 ML steel used for the web of the girder. In order to obtain the required shapes for the puzzle- and clothoid-shaped shear connectors used in the first push-out series, 10 and 20 mm thick steel plates were thermally cut according to EN 1090-1: 2012 [38]. Possible influences from the thermal cutting process as well as from hot-rolling were investigated by tensile tests on dog-bone specimens in accordance with [39]. The German technical approval Z-26.4-56 [24], which forms the basis for the design of the shear connectors used herein, requires that the fabrication of the connector geometry must ensure that no manufacturing-induced defects occur that could cause stress concentrations at the connector edges.

**Figure 3:**
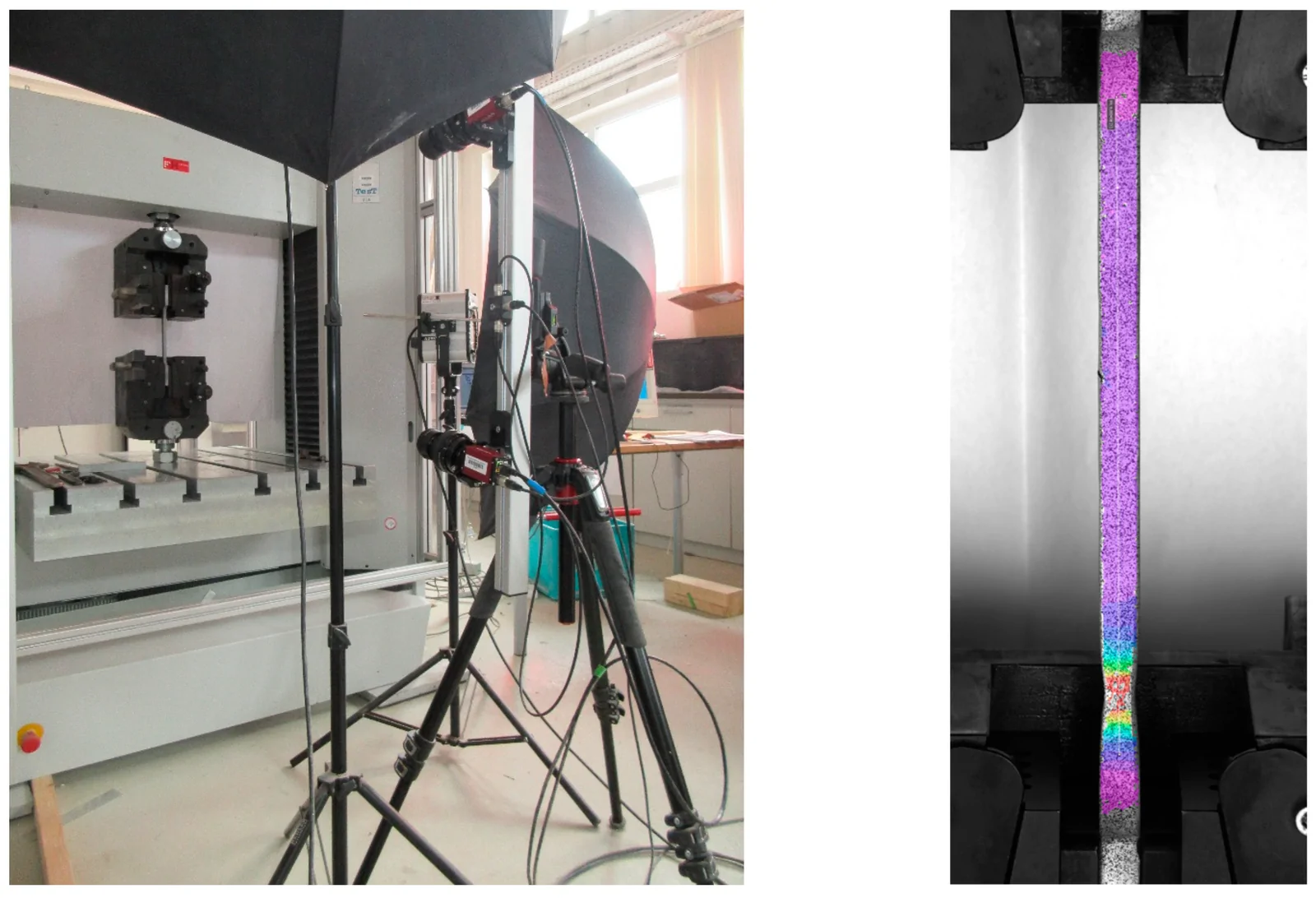
S460 ML steel DIC Test setup (**left**), example of DIC evaluation result (**right**).

The tensile specimens were monitored contact-free using a Digital Image Correlation (DIC) system (Correlated Solutions), which enabled the determination of the complete stress–strain response. For that purpose, a speckle pattern tailored to the size of the measurement field was applied, and the entire loading process was recorded. A detailed description of the procedure used for determining an appropriate DIC setup can be found in [40]. Figure 3 shows the test setup (left) and an example of a DIC evaluation (right) with positioned digital strain gauges and pronounced necking of the steel shortly before fracture.

By comparing the experimentally determined tensile strengths with the values provided by the steel supplier prior to thermal cutting, the manufacturing-related differences could be quantified. The tests showed a reduction in uniaxial tensile strength of 11.93% for the 10 mm plates and 9.22% for the 20 mm plates caused by the thermal cutting process. With average ultimate strengths of 579 MPa (10 mm) and 544 MPa (20 mm), the measured values remain within the allowable strength range for S460 ML steel. No adverse effects associated with manufacturing-induced geometric imperfections were observed in the specimens. An overview of the material properties for the 20 mm plates used in the I-girders is given in Table 2.

Based on the tensile tests evaluated using DIC, an average stress–strain relationship was derived for the 20 mm S460 ML steel used in the girder tests. For implementation in ATENA Science, the curve had to be slightly simplified. The final material law includes the experimentally observed short yielding plateau as well as an approximate descending branch after reaching the maximum tensile stress.

**Figure 4:**
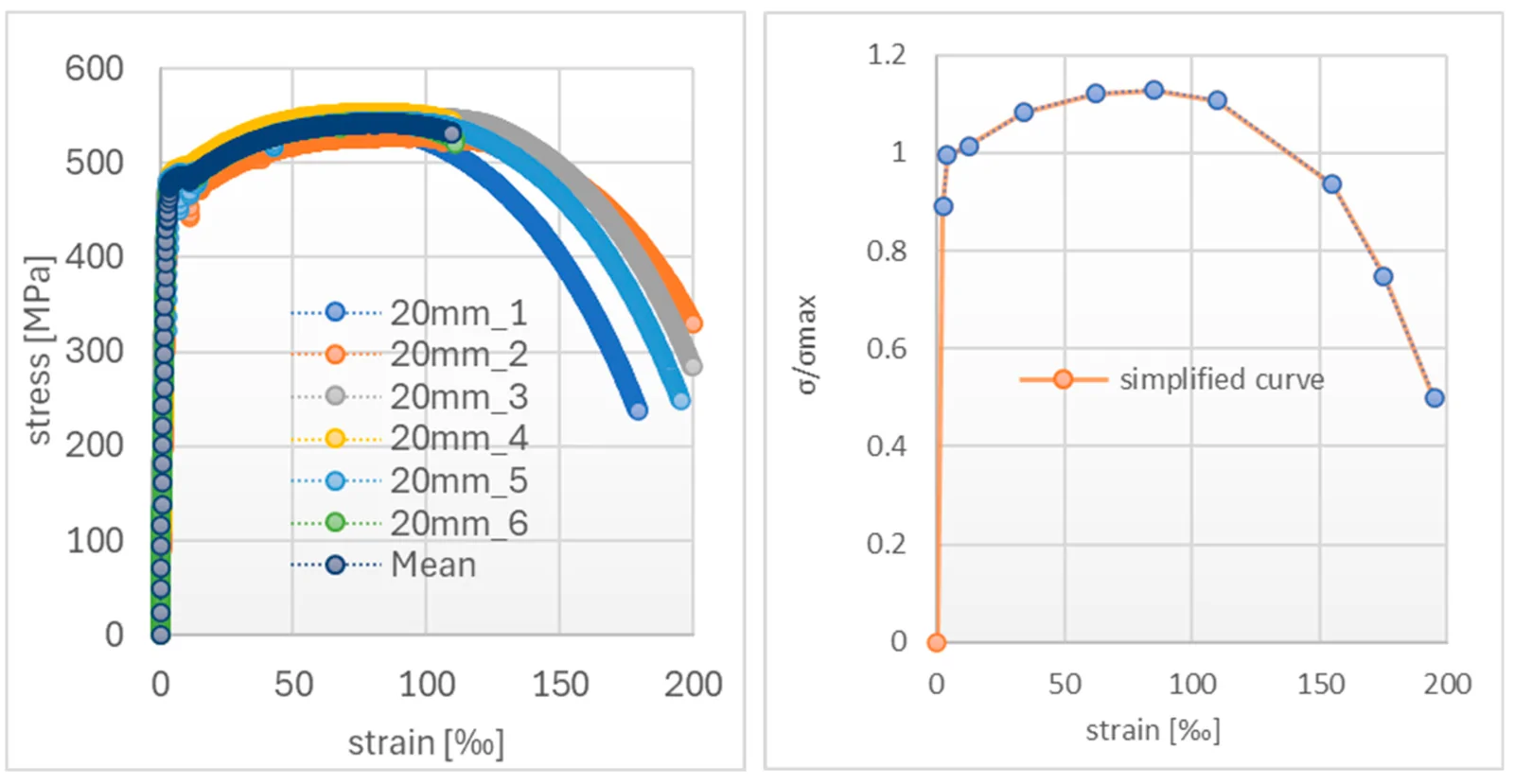
Stress–strain relationship results of S460 ML steel and ATENA Science Input.

ATENA Science provides only a limited bilinear steel material model, which would not allow a direct representation of the measured response. A representation of the measured response—including the short yield plateau and the descending branch after the ultimate stress—requires a multilinear stress–strain law, which the built-in bilinear steel formulation cannot provide. The structural steel was therefore represented by a user-definable constitutive model allowing separate multilinear input of the tensile and compressive stress–strain relations (“Cementitious User 2”). All concrete-specific components of this formulation, namely the shear retention and the tension–compression interaction functions, were deactivated by assigning neutral values. The complete parameter set is given in Appendix A. Figure 4 shows the results of the tensile tests (left) and the averaged stress–strain curve used for the ATENA input (right). This approach was chosen in order to reproduce the experimentally observed tensile behaviour rather than using a simplified bilinear representation.

**Figure 5:**
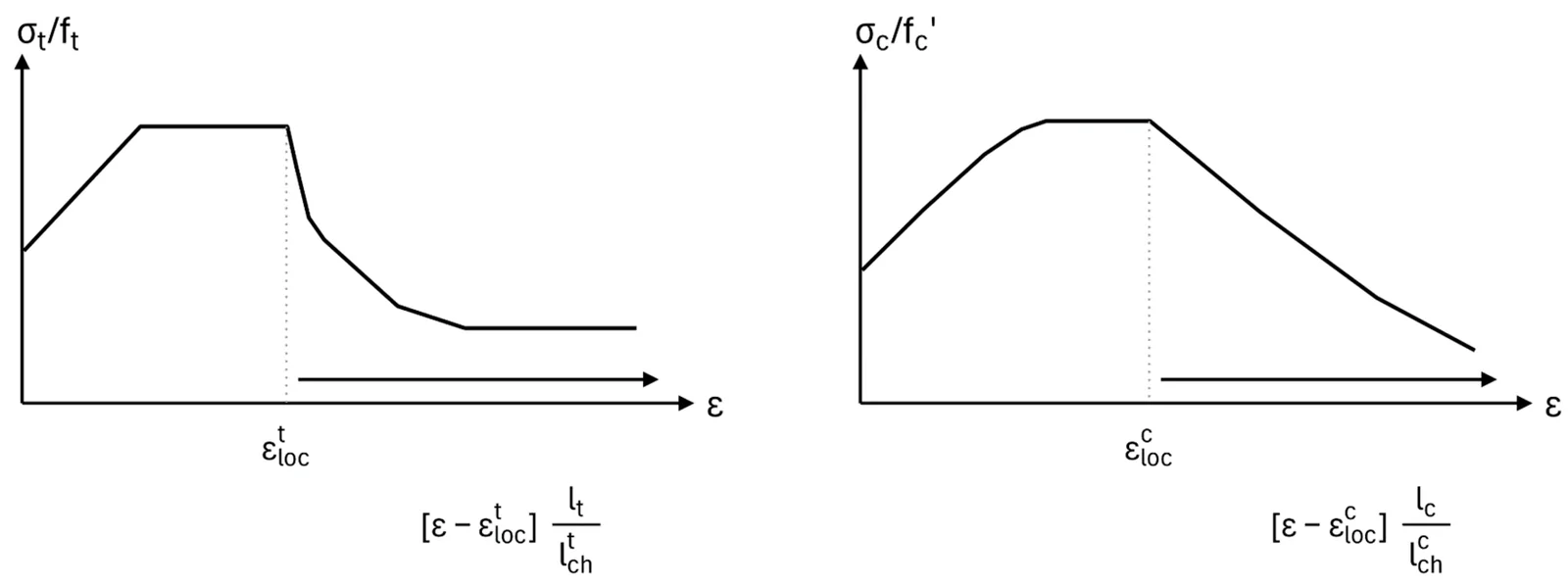
ATENA Science 2025 [41] “cementitious User 2” input relationship for “tensile” (**left**) and “compressive” (**right**).

For implementation in ATENA Science, it should be noted that the stress–strain curves of the steel material must be defined starting from the onset of plastic deformation, where a strain value of zero corresponds to the beginning of the curve. Accordingly, the experimental strain values had to be modified so that the input curve starts at zero strain in ATENA. The general procedure of data input for the material models is also shown in the ATENA Science Input mask as shown in Figure 5.

An overview of all input parameters adopted for the S460 ML material model is provided in Appendix A. The same stress–strain relationship was used for tension and compression of the S460 ML steel.

#### 3.1.2. Prestressing Strand Material Model

ATENA Science provides the option to generate reinforcement material models based on simple input parameters. However, the seven-wire prestressing strands used with immediate bond in the I-girder were not supported by the standard reinforcement library at the time of the numerical study. Therefore, essential input parameters had to be specified manually.

A key aspect of the modelling is the bond–slip behaviour between the prestressing strand and the concrete. ATENA Science offers a default formulation based on the Model Code 2010 [42] using reinforcement-related parameters. A description of the different concrete failure modes and the underlying assumptions for ribbed steel bars in concrete and fibre-reinforced high-performance concrete can be found in [43,44]. However, the embedded bond models are limited to cold-drawn wires and ribbed reinforcement.

Within the research project funded under grant no. 846023, pull-out tests and anchorage tests were carried out for the prestressing strand in combination with the UHPFRC mix used in this study [45]. Anchorage tests were performed within the same experimental campaign to investigate the transfer length of the maximum prestressing force. For the numerical model, it can therefore be conservatively assumed that, in the case of sudden load transfer, the full prestressing force can be introduced into the UHPFRC within a maximum length (l) of approximately 308 mm. Based on a prestressing force of ~200 kN per strand (Fu), divided by the strand diameter d × *π* and the bond length l, this results in an average bond stress τ of 13.15 N/mm^2^ (1), which was determined according to:

(1) $$\tau =\frac{F_{u}}{\pi \times d\times l}$$

Tests on prestressing strands embedded in UHPFRC with comparable compressive and tensile behaviour also demonstrated full anchorage of the strands for an embedment length of approximately 30 × d [46]. Comparable investigations on strands embedded in UHPC report a distinctly non-uniform bond stress distribution along the embedment length and confirm that the transfer length increases considerably once splitting of the surrounding concrete occurs [47]. Numerical studies on the transfer length of pretensioned members further show that the predicted prestress transfer is sensitive to the assumed bond–slip relationship [48]. The assumption of a uniform average bond stress adopted here therefore represents a simplification, which is discussed in Section 5.

This approach for calculating the bond strength assumes that the entire available anchorage length contributes to the bond resistance. In pull-out tests with different embedment lengths, three principal failure modes can occur for bonded prestressing strands: concrete splitting, concrete splitting followed by strand pull-out, and, for sufficiently large embedment lengths, fracture of the strand itself [49]. However, without an extensive test campaign, it is difficult to determine the exact embedment length required to achieve full anchorage of the strands.

The observations from the bond tests performed for the selected prestressing strands are consistent with the findings reported in [46] and serve as a basis for assuming that the bond strength decreases to approximately 80% of its peak value after a slip of about 0.01 m. Slip measurements on the tested I-girders [25] showed maximum strand slips of approximately 0.2 mm up to strand failure, i.e., the strands remained within the fully bonded branch of the adopted bond–slip law throughout the tests.

The experimental campaign reported in [45] further demonstrated that, for the material combination and concrete cover used in this study, full anchorage without concrete splitting failure can be achieved at embedment lengths of 35 d and 45 d.

A relationship between the maximum transferable force per strand of approximately 288 kN (*F*_max_) and the required anchorage length (*l*_max_) is assumed in a first step. Accordingly, the theoretical anchorage length required for full utilisation of the strand up to failure would be determined from the previously obtained anchorage length (l) as a function of *F_u_*:

(2) $$\frac{F_{u}}{F_{\max}}=\frac{l}{l_{\max}}$$

Assuming a simplified linear dependency, this would result in a theoretical anchorage length *l*_max_ of approximately 443 mm for full utilisation of the prestressing strands. Nevertheless, independent investigations by Graybeal [50] indicate that the bond strength does not change in a linear way with the embedment length of reinforcement in UHPFRC.

The bond–slip relationship was consequently derived from the pull-out tests with relatively short embedment lengths. However, the experimental results reported by Vojvodic et al. [45] indicate that this relationship cannot be directly extrapolated to larger embedment lengths. The experimentally determined bond–slip relationship was obtained from pull-out specimens with an embedment length of (10d). Direct application of this local relationship to the substantially longer strand embedment present in the beam would neglect the length dependency of the effective bond response. Vojvodic et al. [45] observed bond-controlled failure for an embedment length of approximately (31d), whereas increasing the embedment length to approximately (47d) resulted in strand failure, indicating that full strand development was achieved within this range.

Accordingly, an effective development length of approximately (40d), representing an intermediate value within the experimentally identified transition range, was adopted as a reference length. The correction factor c applied to the slip coordinates of the experimentally obtained (10d) bond relationship was therefore defined as

(3) $$c=\frac{10d}{40d}=0.25$$

The factor should not be interpreted as a universal material parameter or an exact analytical scaling law. Rather, it provides a physically motivated length-scale correction for transferring the locally measured (10d) pull-out response to the substantially longer, fully developed strand anchorage present in the structural member.

**Figure 6:**
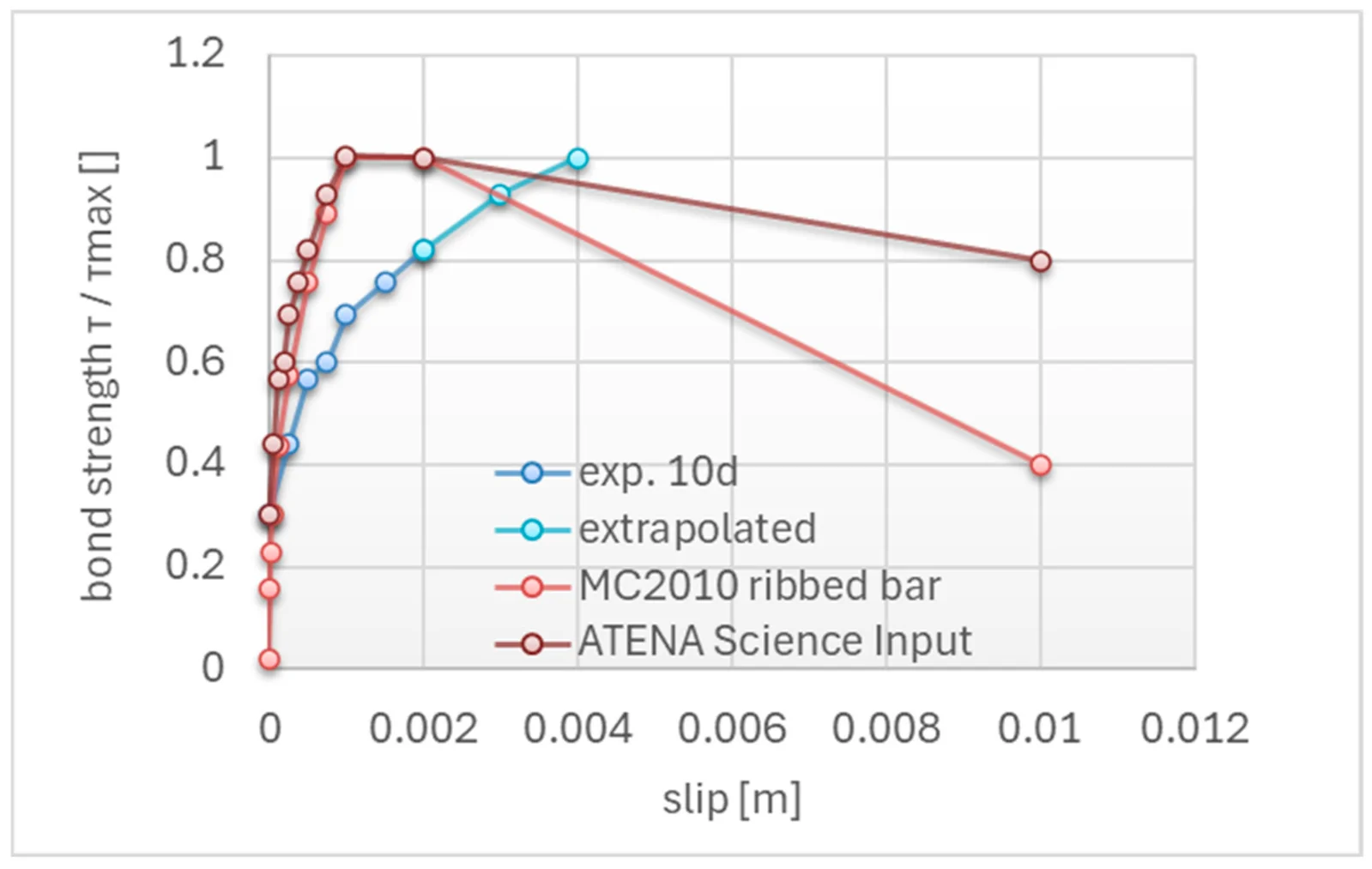
Bond slip relationship—ATENA Science Input.

Based on these measurements and investigations, a bond–slip law was derived by multiplying the experimental and extrapolated slip data by the correction factor c (Figure 6), using the data obtained for an embedment length of 10 times the strand diameter (15.7 mm) published in [45]. For reference, Figure 6 also includes a bond–slip relationship for a ribbed bar enclosed by transverse reinforcement, calculated according to Model Code 2010 [42] for pull-out failure.

**Figure 7:**
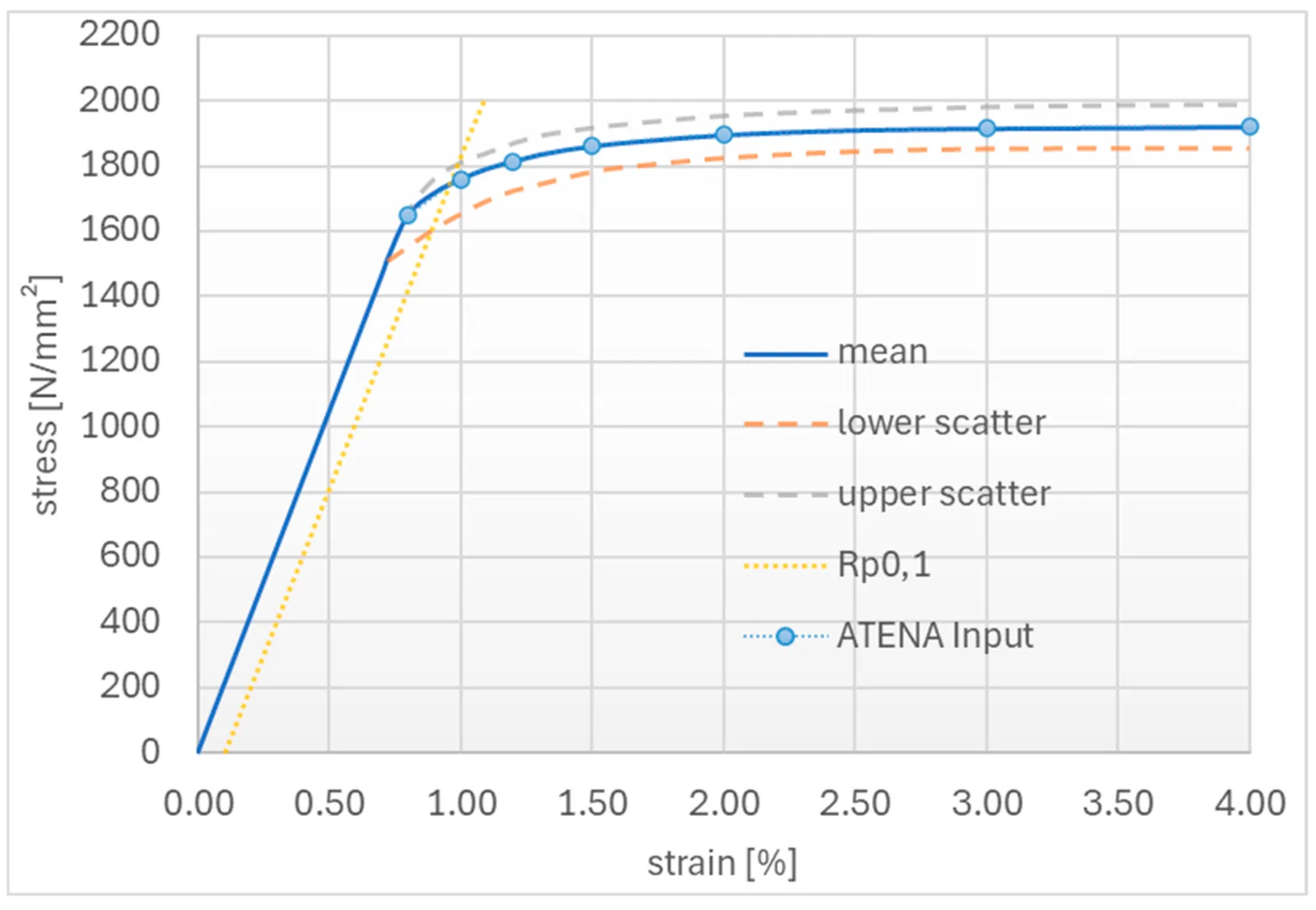
Stress/strain relationship for the prestressing strands—ATENA Science Input.

For the stress–strain relationship of the prestressing strands, the values provided by the manufacturer were used. The corresponding technical approval [51] serves as the basis for the user-defined material law adopted for the strands in Figure 7. Due to the manufacturing process, the ultimate tensile strength of the strands exhibits a certain scatter, since each strand consists of seven wires produced in different production batches. For the plastic range of the material model, the mean value of this scatter was adopted, which is consistent with typical assumptions for prestressing strands when no individual coupon testing is available. An overview of all input parameters is given in Appendix B.

#### 3.1.3. B 550B Material Model

**Figure 8:**
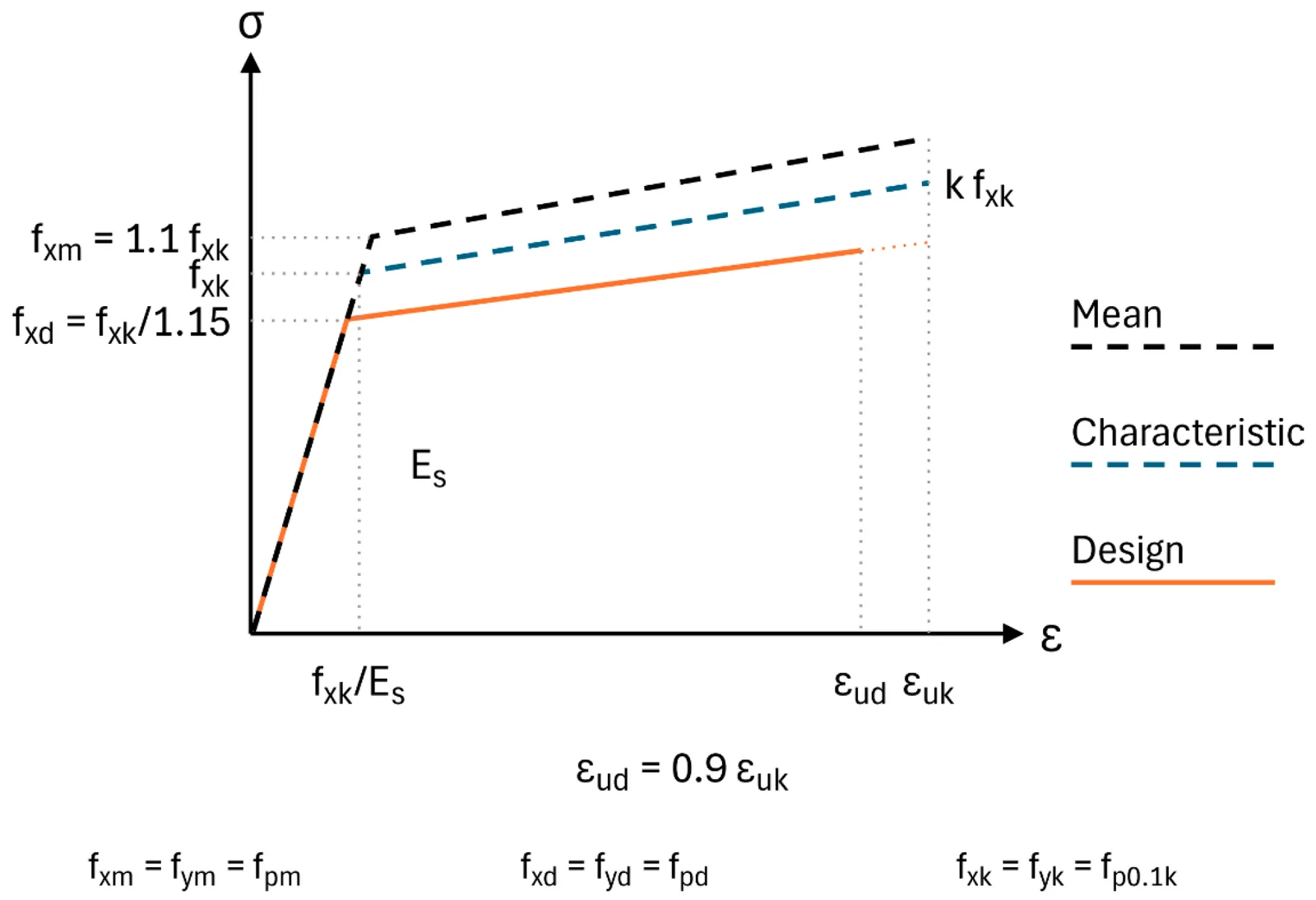
Material model for the conventional steel B550B based on [41].

The material model for the conventional reinforcement in ATENA Science is represented by a bilinear stress–strain relationship. An overview of the material model can be seen in Figure 8. Input parameters were based on publicly available material data for B550B rebars, with a Young’s modulus of 200 GPa, a characteristic yield strength of 550 MPa and a mean yield strength of 605 MPa. The rest of the input parameters were automatically generated by using the “Generate Material” function in the Eurocode 2 (EC2) tab of the reinforcement material model. A more detailed overview of the material input parameters can be found in Appendix C.

#### 3.1.4. Concrete Material Properties

In the present study, the nonlinear behaviour of UHPFRC was represented by the fracture–plastic constitutive model [52] implemented in ATENA Science. The model combines a classical smeared-crack formulation with the crack-band approach according to [53], in which the fracture energy is dissipated over a band width associated with a characteristic element size, thereby limiting the mesh dependency of the softening response. Tensile cracking is governed by a Rankine-type failure criterion followed by exponential softening, applied within either a fixed or a rotating crack formulation. The compressive response is described by a plasticity model with a Menétrey–Willam [54] failure surface, enabling the representation of hardening/softening and confinement effects. This combined formulation allows simultaneous activation of cracking and crushing and accounts for effects such as crack closure under compression. To improve numerical stability under strong softening or dilatancy, the iterative algorithm includes relaxation techniques. Further information on the theoretical background of the concrete material model is given in [52].

##### Tensile Stress-Strain Relationship

For the determination of the uniaxial tensile strength, prisms with dimensions 150 × 150 × 700 mm and accompanying test specimens were tested in a four-point bending configuration according to [55,56]. Crack openings were recorded using a DIC system, which enabled the detection and localisation of each individual crack across the prism width. The suitability of this measurement approach was evaluated in [32] and compared with conventional strain measurements using DD1 strain gauges. The DIC measurements permitted more accurate detection of crack initiation and crack spacing, particularly in the case of small crack distances and at first-crack formation, compared to conventional strain measurement techniques.

The material model was calibrated using a two-dimensional (2D) FE model in ATENA Science on the basis of the four-point bending test (700 × 150 × 150 mm). Since the compressive material properties are of secondary relevance in this test configuration, the setup is well suited for the calibration of the fibre-related and tensile material parameters.

**Figure 9:**
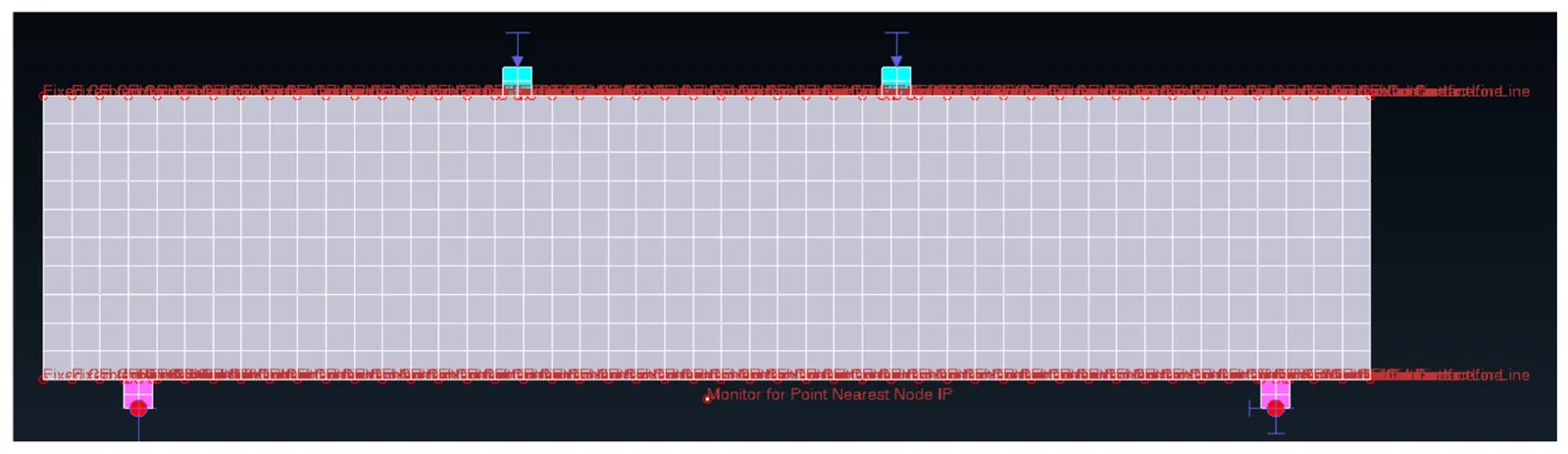
4 Point bending beam, 2D model in ATENA Science.

In the numerical model, simplified master–slave constraints were used for the supports and the load introduction. An overview of the FE model is given in Figure 9.

The simplicity of the geometry and the use of a structured mesh with a width and height of 15 mm allowed for a rapid evaluation of the selected input parameters. The conversion from flexural tensile strength to uniaxial tensile strength (*f_t_*_,*max*_ = 10.64 N/mm^2^) was carried out using the procedure presented in [32].

The input parameters were defined from the physical fibre properties, the concrete strength parameters and the stress–strain relationship obtained by back-analysis of the flexural tests. The characteristic length *l_ch_* is an effective localisation length used to express measured crack opening as an equivalent crack-related strain. It is identified within the inverse-analysis procedure and is not a directly measured width of the localisation zone. The equivalent crack-related strain ε is obtained from the crack opening *w_c_* and *l_ch_*:

(4) $$\varepsilon =\frac{w_{c}}{l_{\mathrm{ch}}}$$

The characteristic length *l_ch_* used for the crack-opening-to-strain conversion is distinct from ATENA’s Tension Characteristic Size, which specifies the reference length of the user-defined tensile stress–strain function used for numerical regularisation. A comprehensive overview of current recommendations for *l_ch_* in existing standards for fibre-reinforced concrete is provided in [57]. A recent systematic comparison of the most common test configurations for deriving the tensile properties of UHPFRC, including uniaxial tests, flexural tests on prisms and on thin plates, evaluated by DIC and inverse analysis, confirms that the derived constitutive law depends markedly on the chosen test setup and evaluation procedure, and provides relationships between crack width and deflection for flexural tests [58]. This underlines the need to identify the characteristic length for the specific material and specimen geometry rather than adopting generic values. The recommended values for *l_ch_* typically range from ½× the specimen depth h to 2 × h, depending on a number of influencing parameters such as fibre length, fibre geometry, tensile strength, anchorage mechanism in the concrete, bond performance of the surrounding matrix, and the geometry of the tested specimen.

A further essential influencing factor for the post-cracking behaviour in the FE simulation is the input parameter regarding crack localisation in the tensile part of the stress–strain law. In order to determine the most appropriate input values for the present UHPFRC with the prototype fibres described above, a parametric study was carried out using the FE model.

The parameter study considered four effective localisation lengths, *l_ch_* = h/4, (h/3), (h/2) and (2h/3), where (h = 150) mm denotes the depth of the 150 × 150 × 700 mm flexural specimens. Each length was combined with three assumptions for the onset of tensile localisation, resulting in 12 FE simulations (Table 3). The resulting tensile relationships were assessed by comparing the simulated flexural load–deflection curves with the experimental mean response.

**Figure 10:**
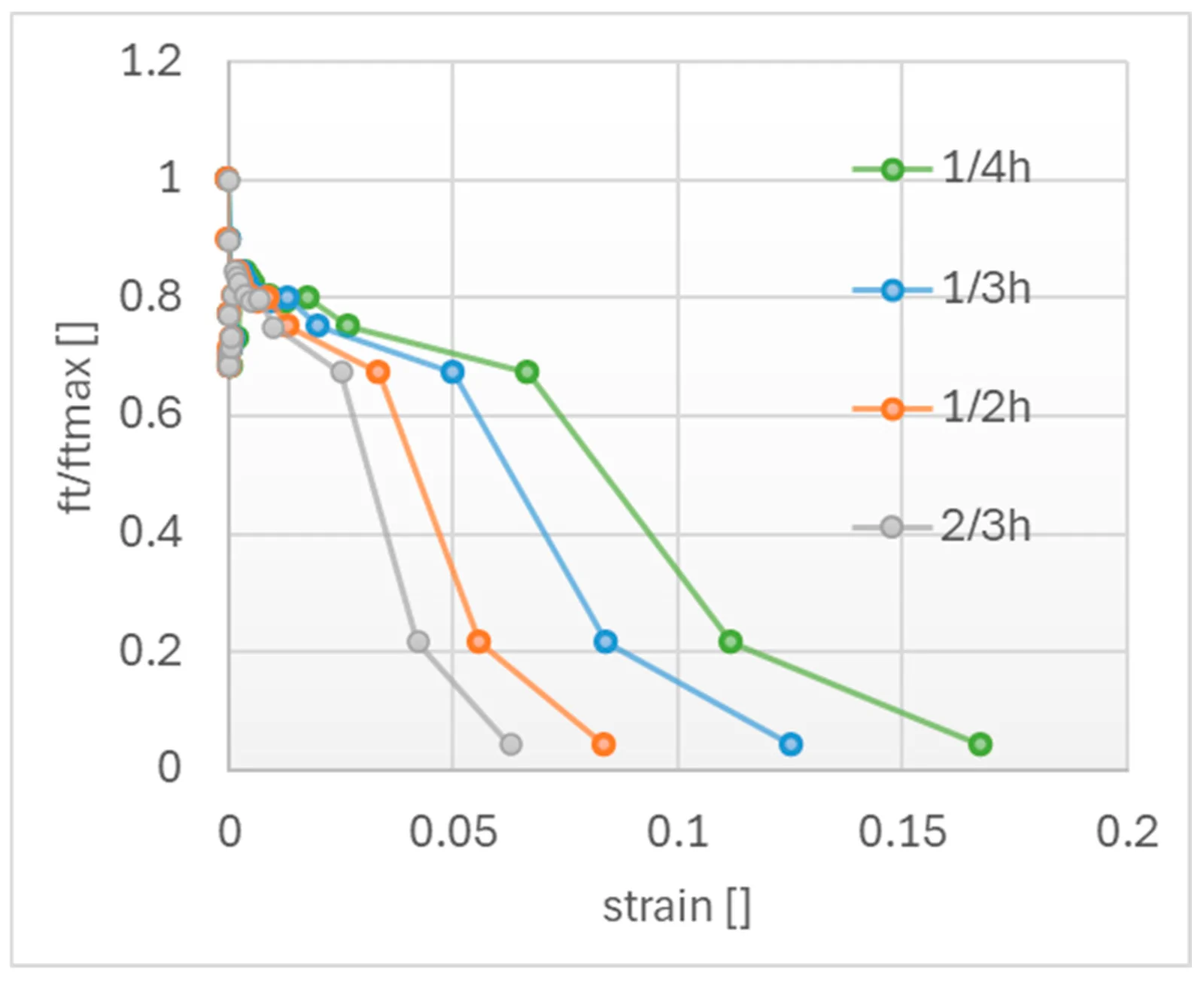
Normalised stress/strain relationships based on the variation of the characteristic length *l_ch_*.

Figure 10 shows the tensile stress–strain relationships obtained using the different values of *l_ch_*. For a given stress–crack-opening relationship, a smaller conversion length produces larger equivalent crack-related strains. This change in strain coordinates does not, by itself, imply an increased physical crack-opening capacity. The latter depends on the fibre-bridging response, including fibre embedment, fibre–matrix bond, pull-out and possible fibre rupture.

A realistic definition of the tensile stress–strain relationship has a significant influence on the cracking behaviour in the concrete in the subsequent FE model, in particular in the region of the shear connectors and the prestressing strands of the prestressed I-girder.

**Figure 11:**
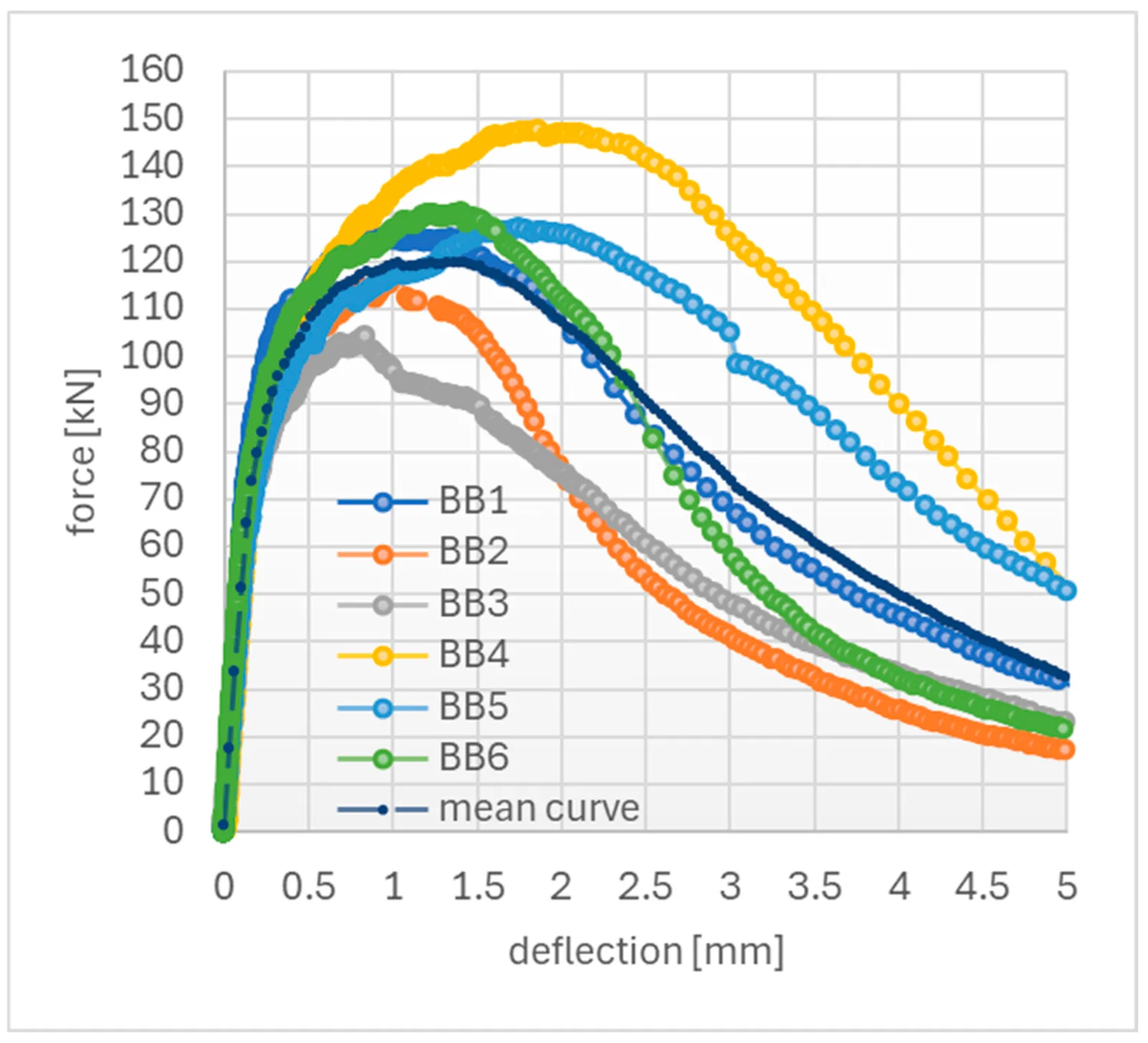
Force/deflection curves of bending beams as a basis for the back analysis.

Figure 11 shows the results of the force–deflection curves for the six experimentally tested specimens (BB1 to BB6) together with the resulting average curve. When interpreting the results, it should be noted that the observable scatter in the experimental data is largely caused by the statistical variation of the fibre distribution, which cannot be neglected.

Since the stress–strain relationship used for the ATENA input was based on the average curve, this curve was adopted as the reference for the parametric study. In the simulations, the fixed-crack option in the “Tensile” tab was deactivated (value set to 0). The “Tension Localisation Onset” parameter in the same tab was defined such that crack localisation was assumed to occur either at the first peak (initial cracking), at the first valley following initial cracking, or at the second peak within the strain-hardening range.

Despite the small maximum aggregate size of only 0.8 mm, the option “Activate Aggregate Interlock” was enabled. Non-quadratic elements were selected for the UHPFRC and the steel web. All relevant input parameters for the FE material model are given in Appendix D.

**Figure 12:**
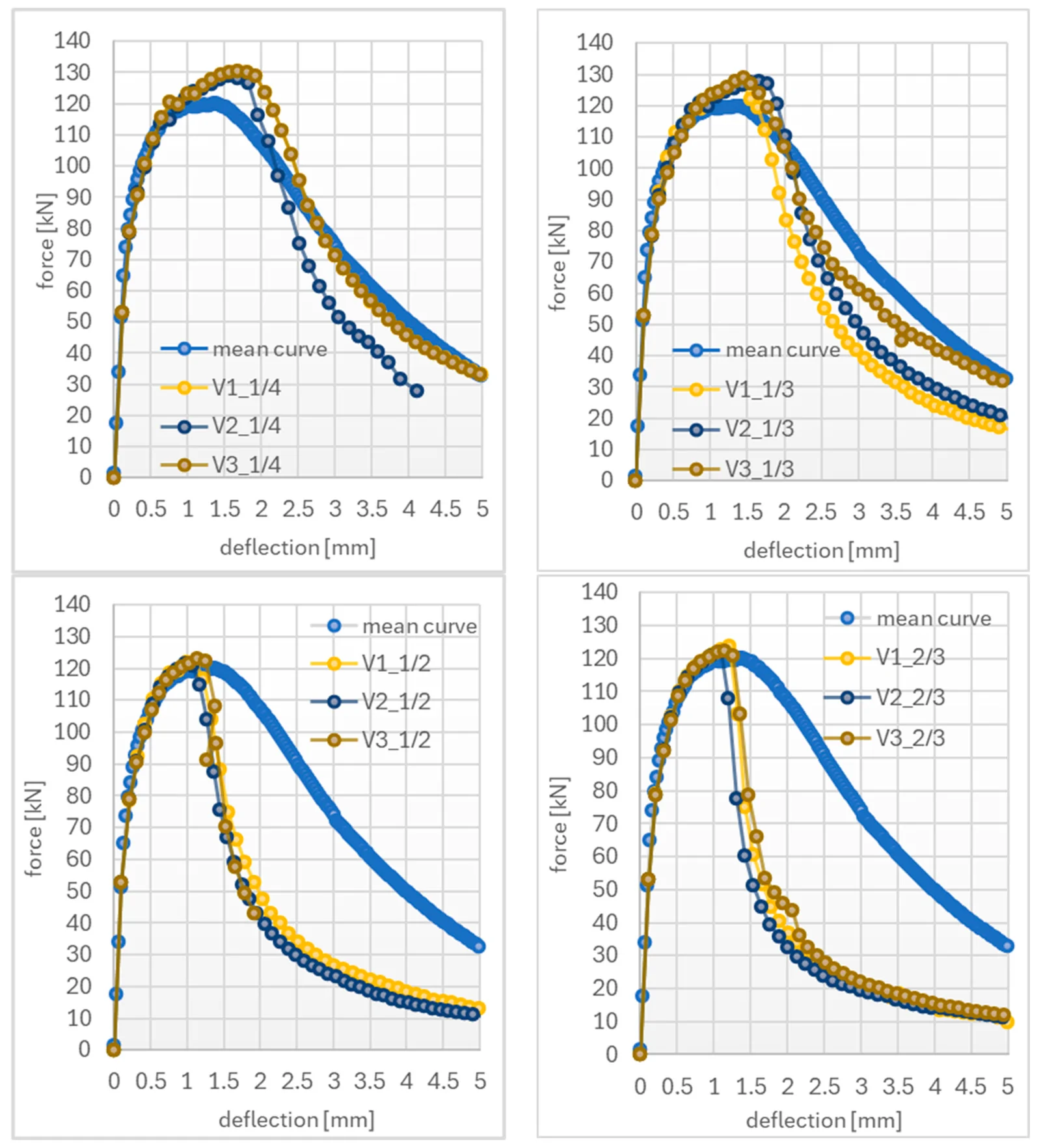
Results of parameter study on the tensile stress/strain relationship.

Among the investigated alternatives, *l_ch_* = h/4 = 37.5 mm provided the closest overall agreement with the experimental mean flexural load–deflection response (Figure 12), with good agreement also observed for specimen BB6. The initial-cracking assumption for the onset of crack localisation was adopted. Larger conversion lengths produced a more pronounced loss of post-cracking resistance. The resulting tensile relationship was therefore retained for the subsequent composite-beam analyses. The identified length is specific to the investigated UHPFRC with its prototype fibre system, the tested flexural specimens and the adopted evaluation procedure; the ratio (h/4) is not proposed as a general relationship for other UHPFRC materials or specimen sizes.

**Figure 13:**
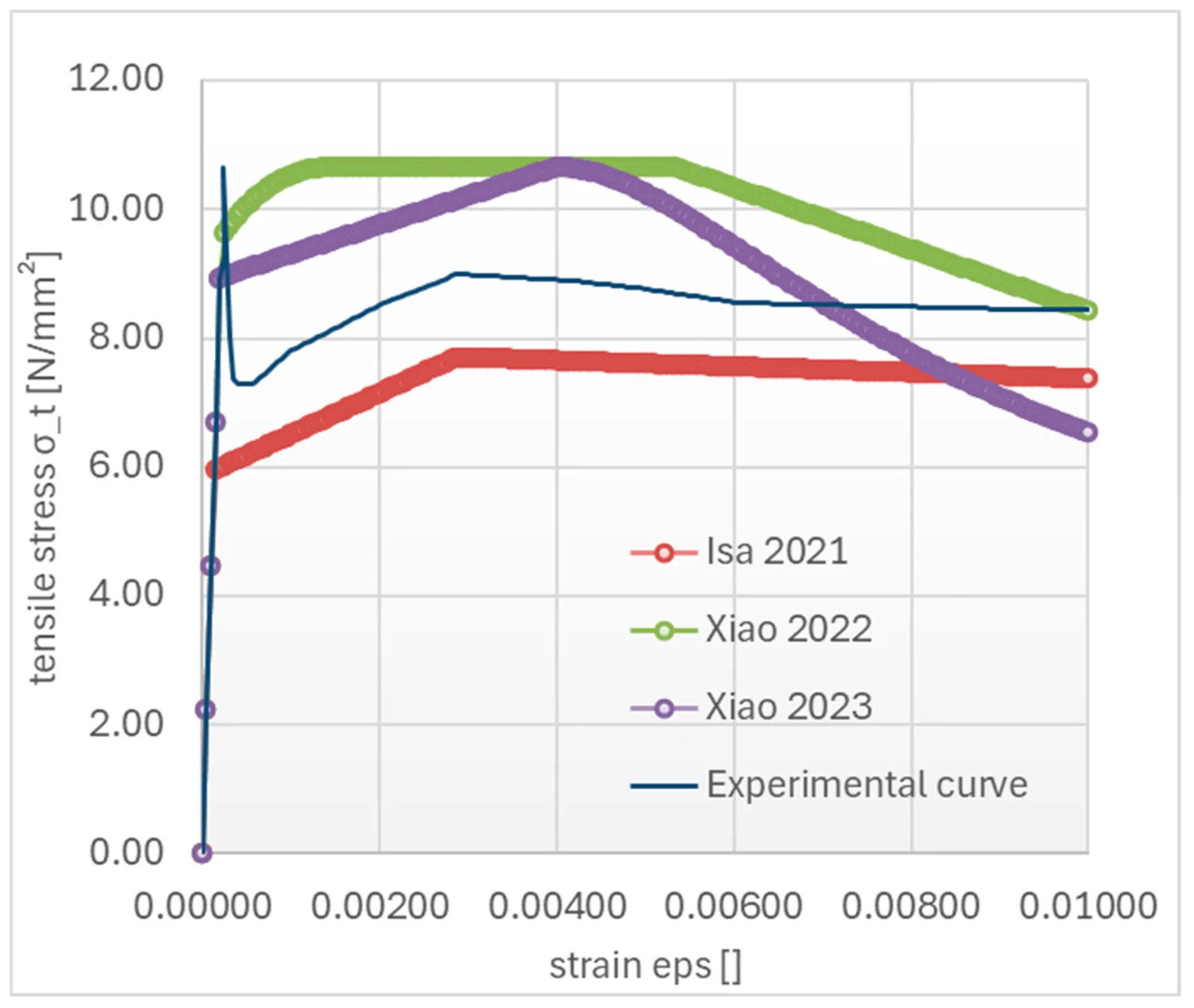
Comparison of experimental tensile stress–strain curve with UHPFRC models; Isa 2021 [59], Xiao 2022 [60] and Xiao 2023 [35].

For an additional assessment of the experimentally derived tensile relationship, the resulting stress–strain response was compared with representative constitutive models available for UHPC. Figure 13 includes the multilinear relationship proposed by Isa et al. [59], the four-stage model proposed by Xiao et al. [60], and the subsequently developed relationship of Xiao et al. [35], together with the constitutive response derived for the UHPFRC investigated in the present study. A systematic description and comparison of these and other commonly used UHPC tensile formulations is provided by Xiao et al. [35]. The analytical formulations are therefore not repeated here. Importantly, the comparison was performed only after completion of the specimen-level calibration and serves as an external plausibility assessment rather than as a basis for selecting or fitting the present material law.

The comparison illustrates the substantial variation in tensile constitutive behaviour reported for UHPFRC, particularly with respect to cracking strength, strain-hardening/softening capacity, strain at maximum tensile stress and post-peak softening. Xiao et al. [35] similarly showed that no single analytical formulation consistently reproduces the tensile envelopes of different UHPC mixtures, reflecting the strong dependence of the response on matrix composition and fibre system. With a maximum tensile stress of approximately 10.6 MPa and a mix of strain-hardening and softening branches, the relationship obtained for the present UHPFRC lies within the range represented by the selected models. The comparison therefore supports the plausibility of the experimentally derived response without introducing literature-based calibration into the subsequent structural analysis.

##### Compressive Stress–Strain Relationship

For the determination of the compressive material parameters, including the post-peak behaviour, an additional displacement-controlled test series was performed for the purpose of additional material characterisation on six 100 × 100 × 100 mm cubes (mean strength 164.8 MPa) and six 200 × 100 mm cylinders (mean strength 151.9 MPa) in accordance with [61]. For the determination of the elastic modulus, three 100 × 100 × 400 mm prisms were tested in accordance with [62]. The specimens were tested using a 5000 kN compression testing machine (FORM + TEST Seidner & Co. GmbH, Riedlingen, Germany), which enabled the measurement of load–displacement curves. In addition, the specimens were monitored from one side, using the DIC measurement system described in the previous sections. Strain measurements for the characterisation of the modulus of elasticity were taken using DD1 strain gauges (Hottinger Brüel & Kjaer, Darmstadt, Germany). The material characterisation yielded an average secant modulus of 44.6 GPa.

**Figure 14:**
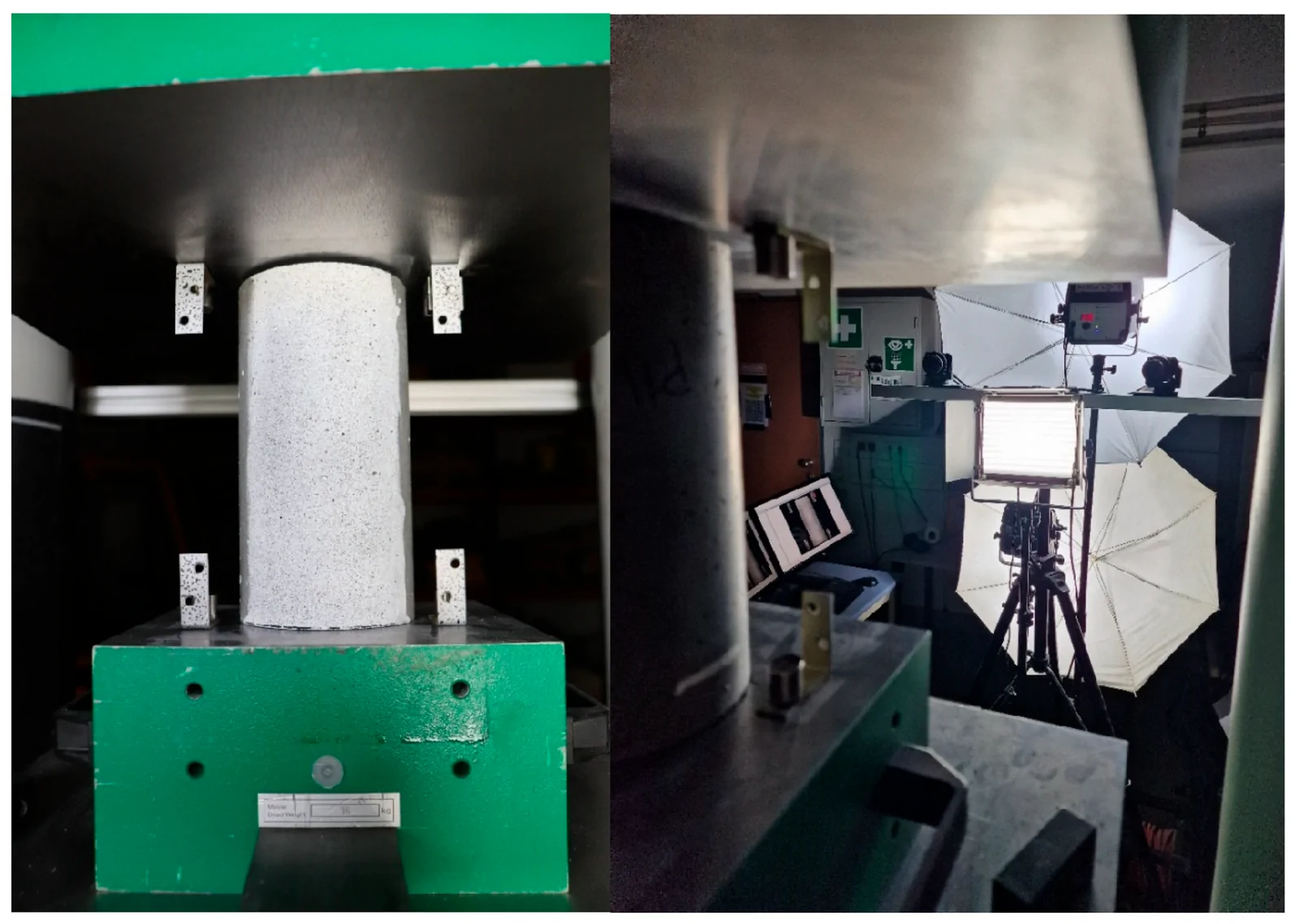
Test layout of compression tests.

Figure 14 shows the test setup and the measurement equipment, including the applied speckle pattern and the positioned measurement markers. To capture the post-peak deformation behaviour, the tests were performed in displacement control at a rate of 0.3 mm/min.

Based on the conducted tests, the stress–strain relationships can be derived using the specimen dimensions and the measured deformations. However, due to the nonlinear stress distribution across the section in cube compression tests, the resulting compressive stress–strain curve must be further processed before it can be used in the material model. An overview of the different stress distributions depending on specimen geometry is provided in [63]. In cube tests, the restricted lateral expansion beneath the loading plates leads to a triaxial stress state, whereas this effect is significantly less pronounced in cylinders due to their axisymmetric geometry.

The DIC System could only be utilised up to the onset of plastic deformation. The typical hourglass-shaped failure mode of cubes under compression makes it virtually impossible to derive representative surface strains for the compressive stress–strain law from that point, as spalling of the outer shell causes the recorded strains to no longer reflect the behaviour of the full cross-section. The inclined cracking observed in the cylinders after peak stress also complicates the extraction of representative strains, as friction, interlocking, crack inclination and other effects lead to a highly localised and non-uniform stress field. Other means of measuring the post-peak plastic behaviour, such as the use of DD1 strain transducers, were ruled out due to the significant danger of destruction of the sensors due to sudden brittle failure at peak or to large deformations in the plastic part of the tests.

The strain measurements recorded over the full specimen height—affected by the aforementioned mechanisms—combined with elastic deformations of the loading plates, made it necessary to determine the compressive strain from the machine displacement instead. This is possible by applying a correction factor that accounts for the actual stiffness of the UHPFRC. Reference [64] introduces a procedure for data correction/smoothing and a correction factor *n_E_* (5), defined as the ratio between the apparent modulus obtained from cube or cylinder tests and the elastic modulus obtained from prism tests. All effects such as end restraint, machine deformation, and local elastic penetration/deformation of the steel plates cause the modulus of elasticity derived from the cube tests—using the average stress–strain curve computed from the applied load and machine displacement—to be systematically underestimated.

(5) $$n_{E}=E_{\mathrm{cube},\mathrm{cyl}}/E_{\mathrm{cm}}$$

In Equation (5), *E_cube,cyl_* denotes the apparent elastic modulus derived from the mean cube or cylinder response using machine displacement, and *E_cm_* denotes the mean secant modulus measured on the prisms.

The initial branch of each mean compressive stress–strain curve was first linearised. The strain coordinates of the resulting curve were then corrected pointwise using Equation (6), while the corresponding stress values were retained. Here, *ε_mean_* denotes the strain of the mean curve after initial linearisation, and *ε_corr_* denotes the corrected strain. The procedure was applied separately to cubes and cylinders using the respective coefficient *n_E_* from Equation (5).

(6) $$\varepsilon_{\mathrm{corr}}=n_{E}\;\cdot \;\varepsilon_{\mathrm{mean}}$$

**Figure 15:**
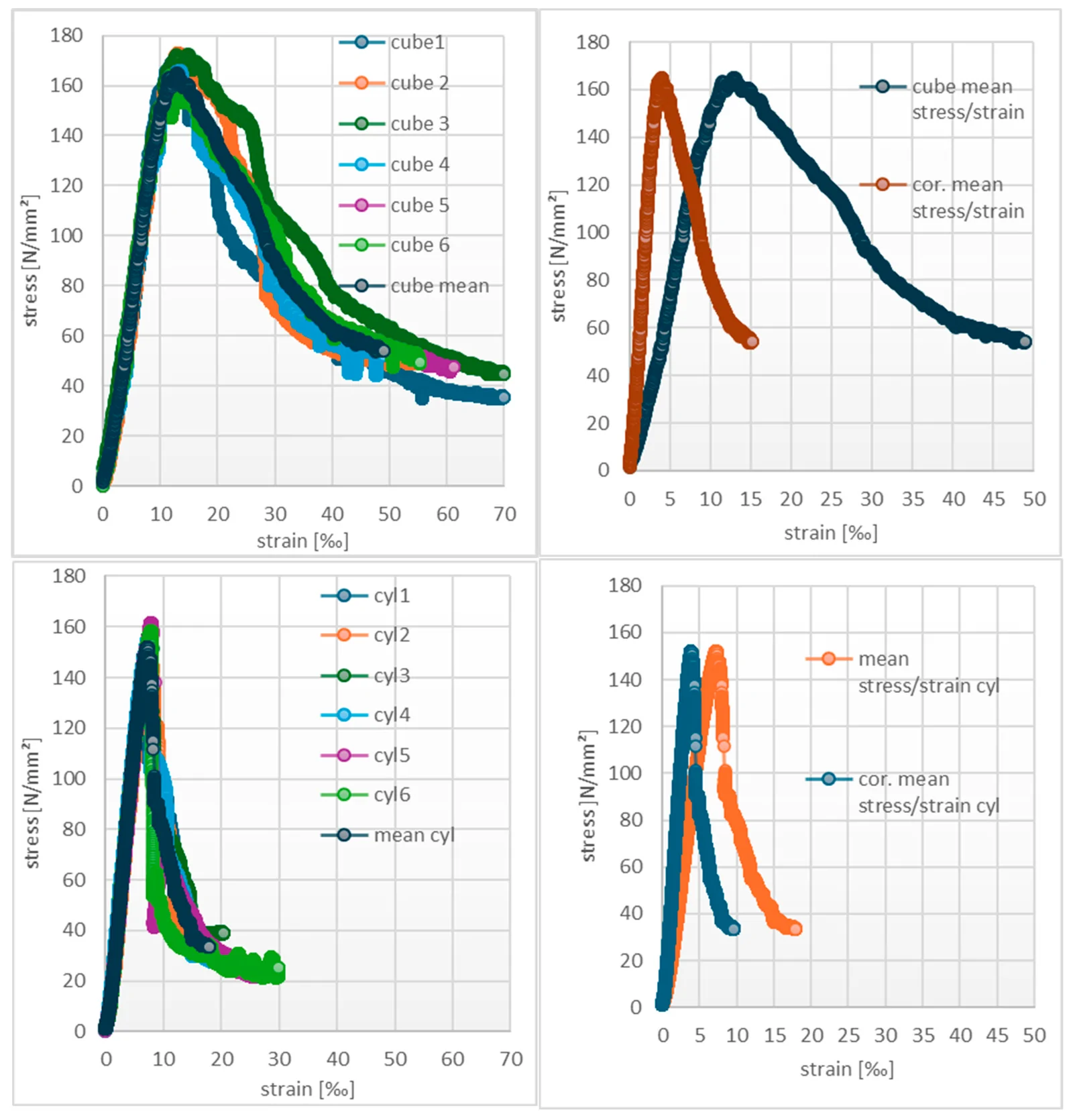
Stress/Strain relationship from cube and cylinder compressive tests.

For the cube tests, an apparent elastic modulus of 13,782 N/mm^2^ was obtained, resulting in a correction factor of ~0.309. For the cylinders, the apparent elastic modulus was 23,851 N/mm^2,^ corresponding to a correction factor of ~0.535. Figure 15 shows the results of the compression tests on cubes and cylinders, the respective average curves, and the converted stress–strain relationships based on the corresponding correction factors.

The resulting compressive stress–strain curve derived from the cube tests shows good agreement with published experimental campaigns on UHPFRC mixtures of similar composition but with different aggregate gradations and fibre contents (see e.g., [65,66]). After applying the correction factor *n_E_* the maximum compressive stress is reached at a strain of approximately 3.8‰, followed by a pronounced stress drop. With further loading, a more gradual decrease in stress is observed, which can be attributed to the progressive mobilisation of steel fibres under a triaxial stress state in the central region of the cube.

A significantly different behaviour is observed in the data from the cylinder tests. Due to the reduced confinement beneath the loading plates and a comparable level of machine deformation and applied load, the resulting stress–strain curves exhibit markedly different characteristics. In contrast to the cube tests, the cylinders show a sudden drop in load-carrying capacity immediately after the peak stress, followed by a substantially lower plastic deformation capacity. This behaviour is consistent with the expected transition from a confinement-driven post-peak response in cubes to a predominantly uniaxial failure mechanism in cylinders.

The proposed approach can be further verified by normalising the derived stress–strain relationships. In addition to the main test campaign, the three prisms used for the Young’s modulus tests were loaded to failure under displacement control after completion of the modulus measurements and included in the evaluation. The aim was to determine whether the stress–strain curves derived using the calculated correction factors show consistent behaviour across different specimen geometries and associated stress states.

**Figure 16:**
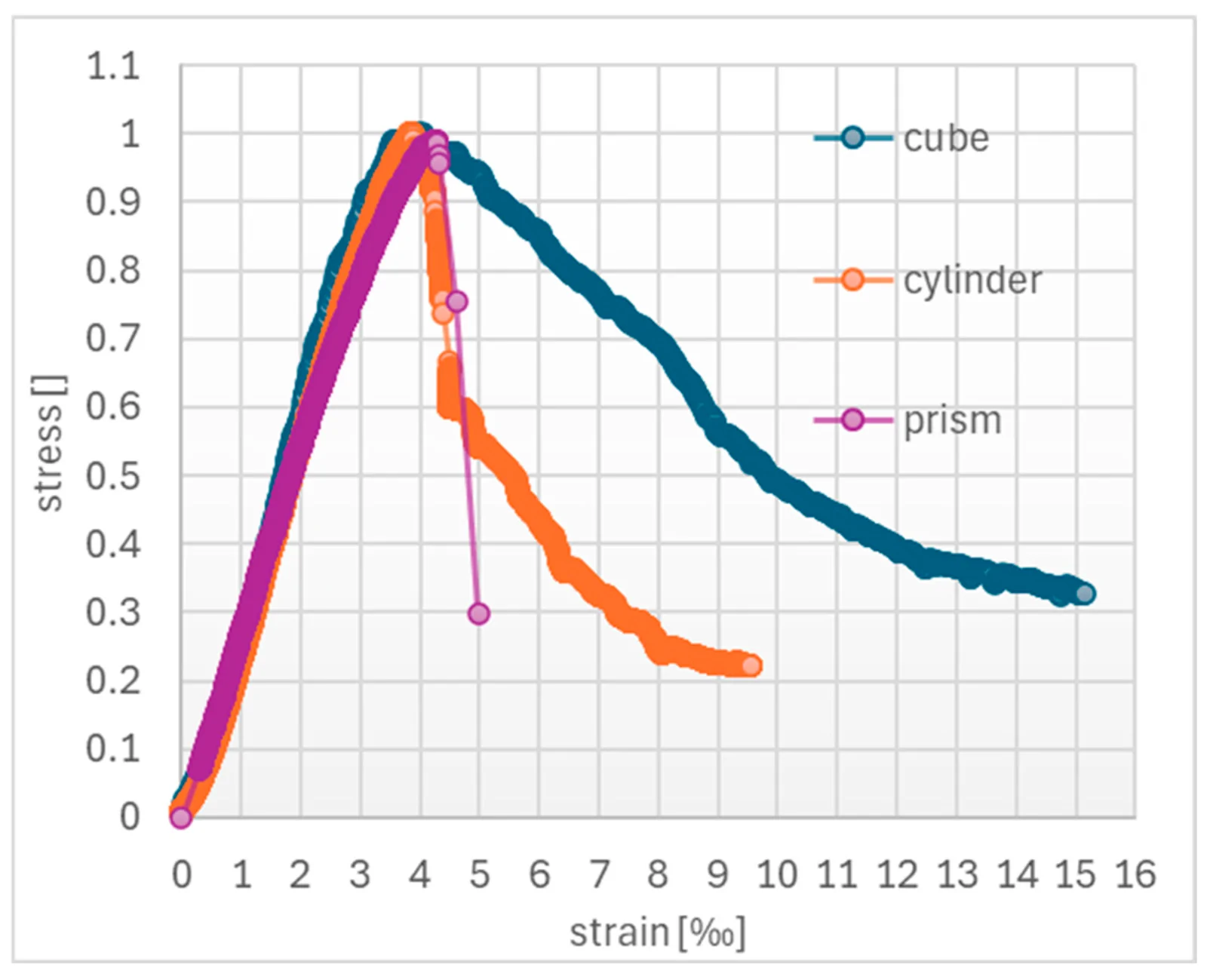
Normalised comparison of derived cube, cylinder and prism stress/strain-relationships.

For this purpose, the stresses of the respective mean stress–strain curves were divided by their individual peak stresses. Figure 16 shows the resulting normalised stress–strain curves, illustrating the degree of correlation between cube, cylinder and prism specimens.

The cylinder-shaped specimens exhibited a sudden and brittle failure, which prevented the development of a representative plastic branch in their stress–strain relationship. Across all test series, it became evident that the confinement effects in the cube specimens, caused by frictional restraint under the loading platens, resulted in a significantly greater deformation capacity compared with the other specimen geometries. In contrast, the long prismatic specimens with a height of 400 mm—where the stress state is only minimally influenced by lateral restraint—failed abruptly and with very limited ductility, showing a rapid post-peak drop to approximately 30% of the peak stress. The cube specimens, however, sustained more than 10‰ additional strain before reaching a similar stress level, owing to the triaxial compression state that develops in their core.

**Figure 17:**
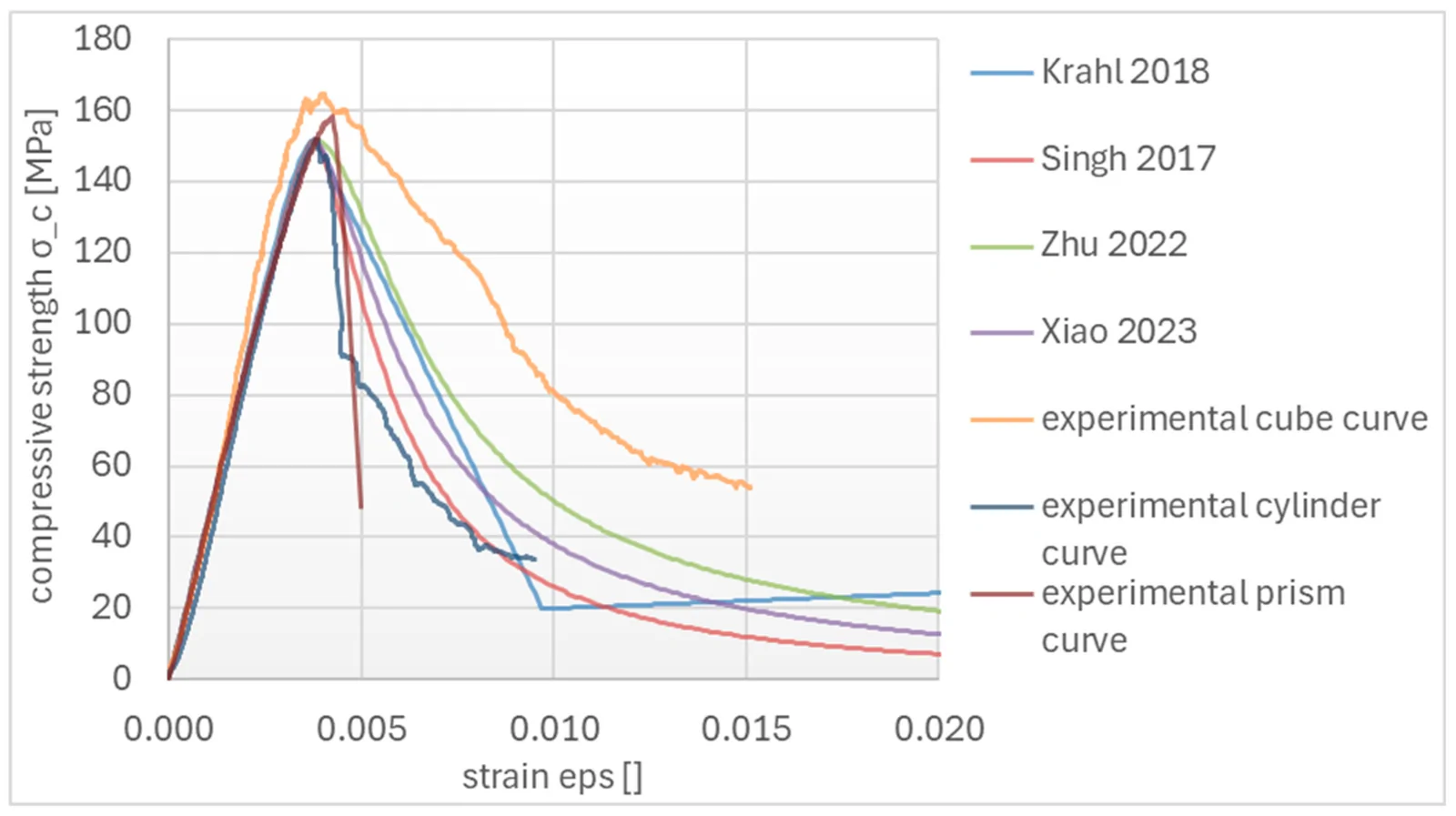
Comparison of UHPFRC stress-strain models for compression with experimental results; Krahl [67], Singh 2017 [68], Zhu 2022 [69] and Xiao 2023 [35].

For an additional assessment of the experimentally derived compressive response, the corrected stress–strain relationships were compared with representative analytical UHPFRC models from the literature. Figure 17 compares the present cube- and cylinder-derived curves with the constitutive relationships proposed by Krahl et al. [67], Singh et al. [68], Zhu et al. [69] and Xiao et al. [35]. These models represent different approaches to describing the ascending and descending branches of UHPC under compression and are systematically reviewed by Xiao et al. [35]. The original publications are cited as the sources of the individual relationships, while the overview by Xiao et al. is used for their comparative context.

The comparison shows that the ascending branches and the strain at peak stress of the experimentally derived curves are generally consistent with the range represented by the published models. More pronounced differences occur in the post-peak response. The cylinder-derived curve approaches the comparatively rapid strength degradation predicted by the literature relationships, whereas the cube-derived curve retains a substantially higher residual compressive stress with increasing strain. This difference is consistent with the experimentally observed influence of specimen geometry and lateral restraint discussed above. The comparison therefore supports the plausibility of the applied correction procedure while simultaneously demonstrating that the extended post-peak deformation capacity of the cube specimens should be interpreted as a confinement-influenced response rather than as a purely uniaxial material characteristic.

**Figure 18:**
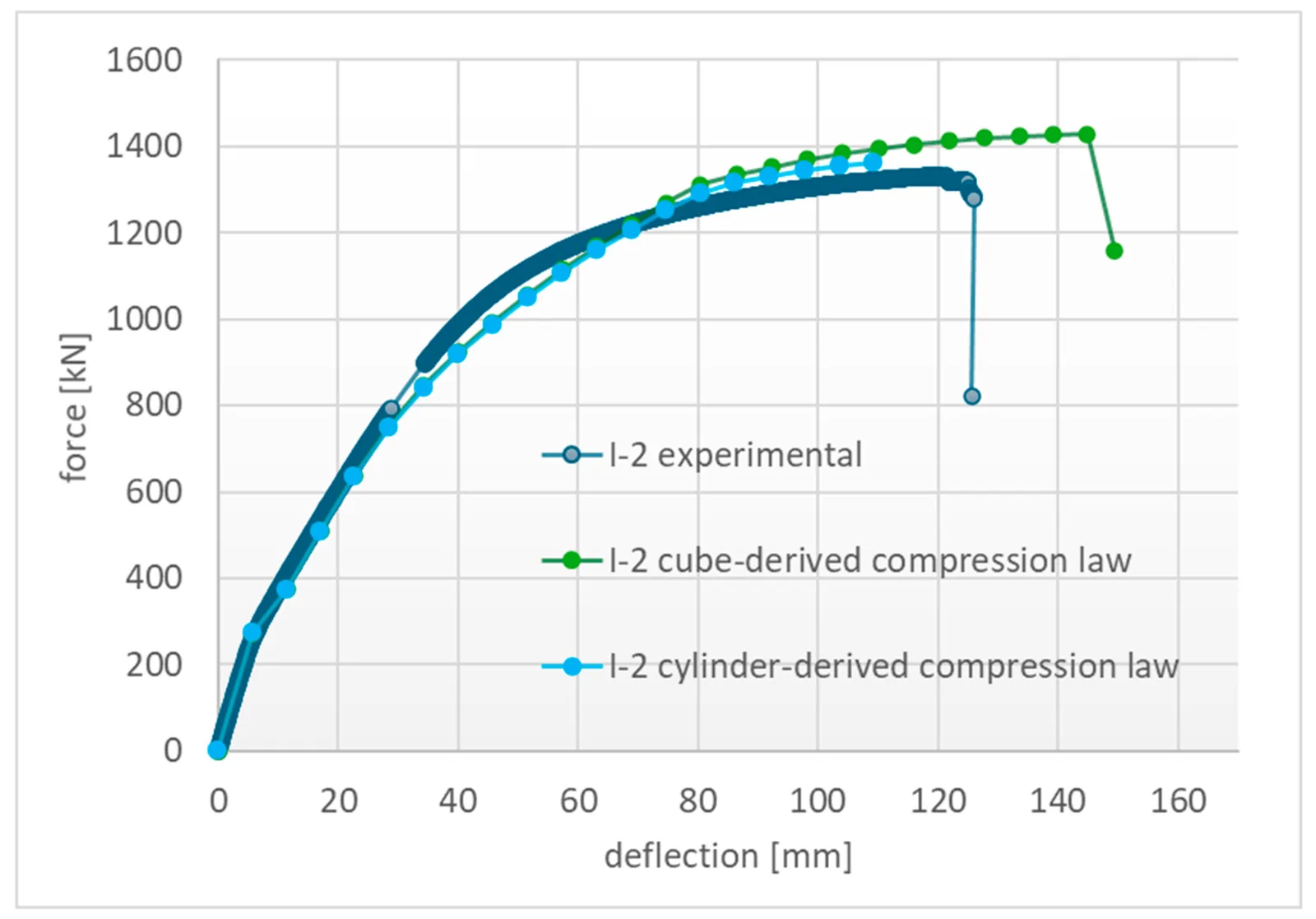
Preliminary cube-versus-cylinder compression-law comparison for I-2 at the original 35 mm mesh and 35 mm Tension Characteristic Size.

As a preliminary sensitivity check, I-2 was analysed using the cube- and cylinder-derived compressive relationships at the original reference settings (35 mm mesh and 35 mm Tension Characteristic Size; Figure 18). The cylinder-based analysis reached approximately 1360.9 kN at 109.3 mm and terminated before the strand-rupture state, whereas the cube-based analysis reached approximately 1428.9 kN at 144.9 mm and continued to strand-controlled failure. This comparison informed selection of the cube-derived relationship as an effective input for the present framework. It predates the additional tensile reference-size study in Section 4.1 and is not a controlled compression-law comparison at the final 25 mm Tension Characteristic Size.

This comparison is not interpreted as evidence that the cube-derived relationship represents a general uniaxial material law; rather, it indicates that its increased post-peak deformation capacity provides a more suitable effective representation of the compressive response within the restrained regions of the investigated composite beam. For the prestressed composite I-girder considered later in this study, the presence of prestressing strands, conventional reinforcement and shear connectors indicates that a certain level of confinement and restraint against lateral expansion will also be present. The previously conducted push-out test campaign [23], which identified the governing failure mechanisms in the region of the puzzle-shaped connectors, further suggests that the compressive behaviour of the UHPFRC in the girders will more closely resemble that of the restrained cube specimens than that of cylinders or prisms. The compressive constitutive relationship derived from the cube tests was therefore retained consistently throughout the numerical study, as it allowed the complete failure sequence, including strand rupture and post-peak redistribution, to be reproduced. The resulting response was considered appropriate for the present modelling approach, in which several local stiffness contributions of the slender composite web, including reinforcement- and connector-related effects, are necessarily represented in a simplified manner. The cube-versus-cylinder comparison is a member-level model-selection step. The retained curve shape was not subsequently refitted to the beam response; selection of the tensile material-reference size is reported separately in Section 4.1. This choice does not establish a unique representation of confinement or exclude overlap between specimen restraint in the input curve and multiaxial effects in the constitutive formulation.

**Figure 19:**
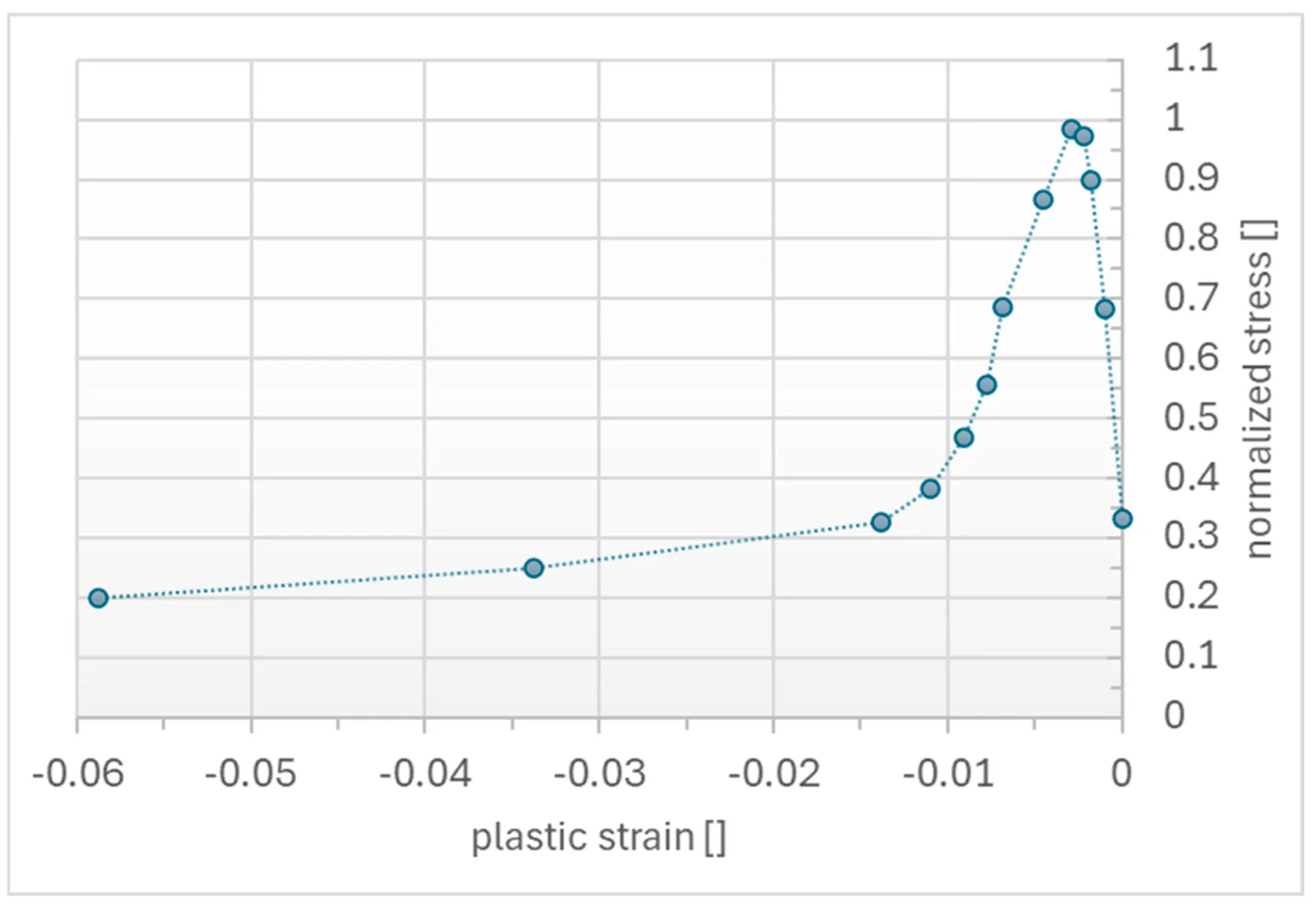
UHPFRC stress–strain relationship in compression.

For the material model in ATENA Science, the curve derived from the cube tests was further processed and simplified. Figure 19 shows the final material model for the plastic compression part of the concrete model.

For the implementation of the user-defined “Cementitious2 SHCC/HPFRC” material in ATENA Science, where SHCC denotes strain-hardening cementitious composites, and HPFRC denotes high-performance fibre-reinforced concrete, it was necessary to specify additional input parameters beyond the tensile and compressive stress–strain relationships. These parameters relate primarily to the geometry and mechanical characteristics of the steel fibres, which are required in the “Fibre Reinforcement” tab to correctly represent the contribution of fibre action in tension and can be found in Appendix D.

In summary, the tensile and compressive parts of the UHPFRC constitutive model are directly calibrated against dedicated material tests, taking into account the effects of confinement, machine deformation and crack localisation. The final stress–strain relationships adopted in ATENA therefore represent an experimentally supported modelling approximation for the UHPFRC response in the critical regions of the present composite beams.

### 3.2. Finite Element Model of the Composite Beams

The calibrated material models described in the previous sections were subsequently implemented in the finite element model of the prestressed UHPFRC I-beam. Exploiting geometric symmetry, only half of the beam was modelled along its longitudinal axis (half widths), reducing computational effort.

**Figure 20:**
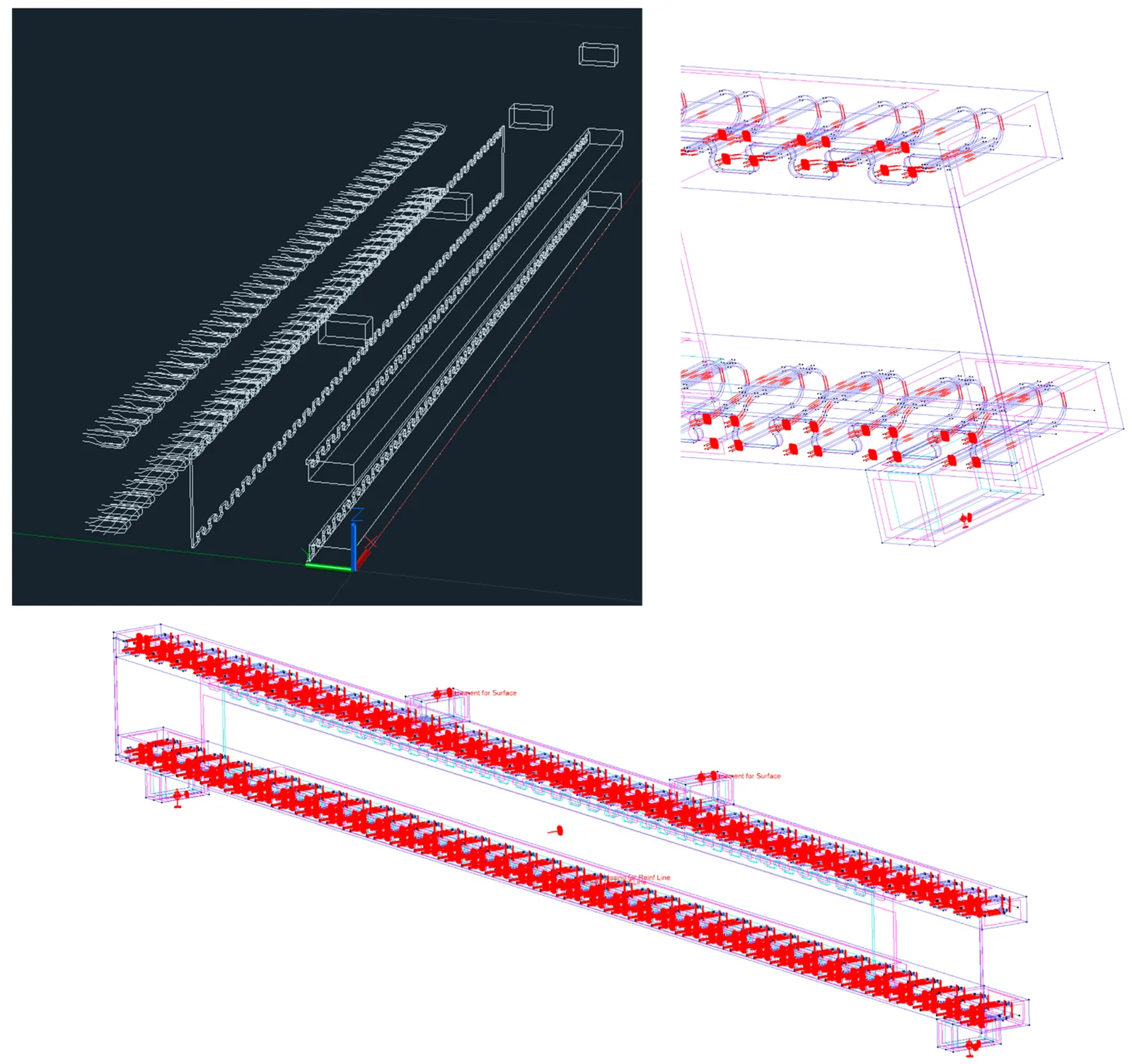
3D model from CAD (top-left); GiD 17.0.1 model with boundary conditions after moving the elements (**bottom**) and detail (**top-right**).

The beam geometry, including the puzzle-shaped shear connectors and one-dimensional (1D) reinforcement paths, was prepared in 3D and imported into GiD 17.0.1 using Drawing Exchange Format (DXF) (Figure 20, top).

Volumes and reinforcement paths were imported as separate entities and assembled into the half-beam model to avoid geometric conflicts.

Figure 20 (bottom) shows the final FE geometry together with the applied boundary conditions, including:

- Horizontal restraints of the steel web;
- Support conditions replicating the experimental setup;
- Constraints along the symmetry plane, where the 1D reinforcement elements were fixed to ensure correct symmetry behaviour.

**Figure 21:**
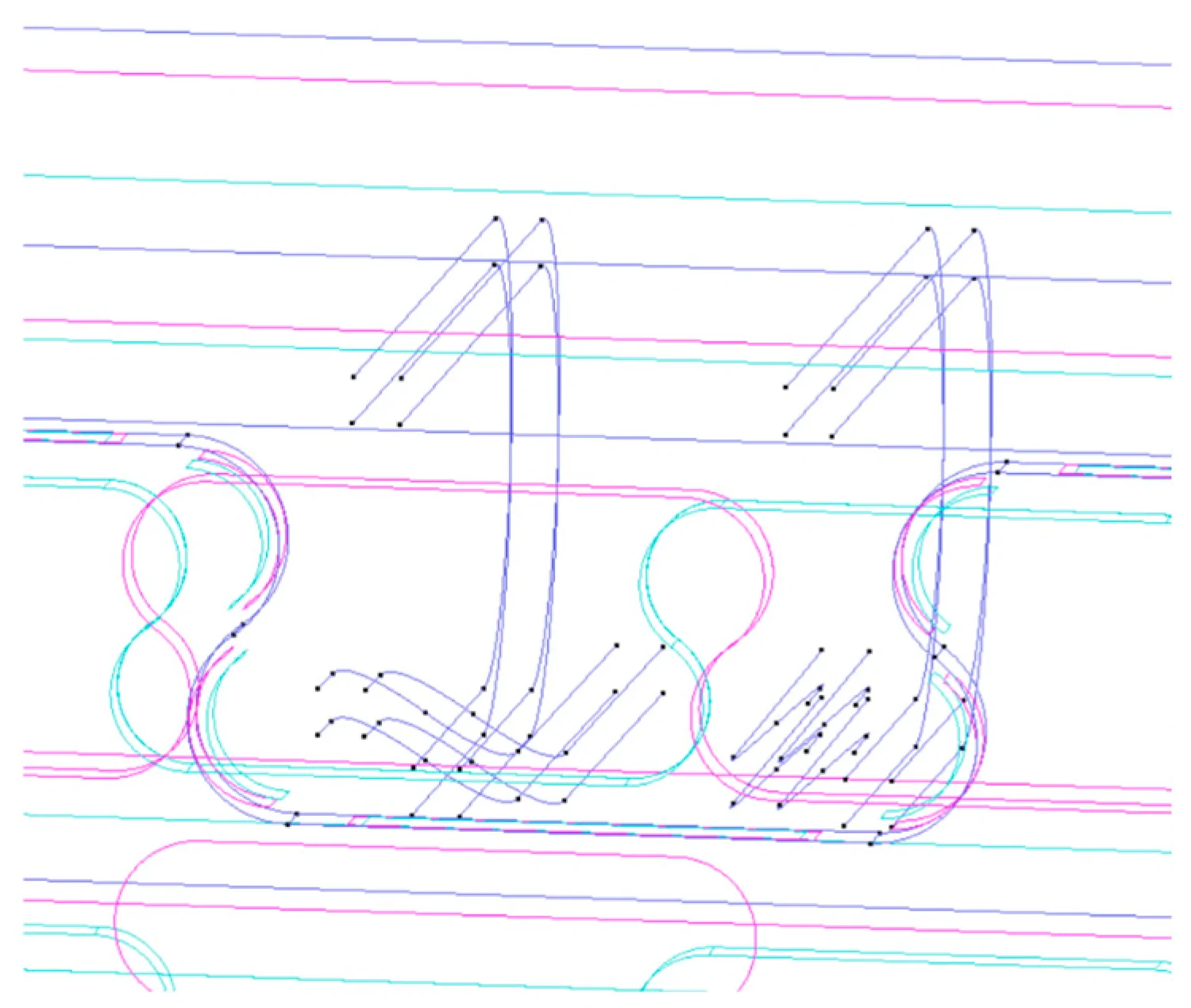
Reinforcement detail for B550B in the FE model.

A key modelling aspect for the B550B reinforcement is illustrated in Figure 21, showing four parallel reinforcement paths representing a single B550B bar in the model. Each path was assigned one quarter of the actual bar cross-sectional area, preserving the total steel area and axial stiffness. The B550B elements use ATENA’s built-in bond–slip model for conventional reinforcing steel. The subdivision distributes the reinforcement stiffness spatially, but does not preserve the bond perimeter of a single physical bar. The larger summed perimeter may therefore overestimate local bond transfer. This approximation is retained for the present assessment of global beam response and internal stiffness distribution. The prestressing strands use the separate user-defined bond–slip law described in Section 3.1.2.

The Arc-length method in combination with the “Pardiso” solver and activated line search method was selected for the solution parameters. The imposed displacements were applied using surface displacement boundary conditions, with a displacement of 0.005 m, an “Interval Multiplier” of 140 and a “Number of Load Steps” of 140. The loading and support plates were modelled using the “solid elastic” material type. Interface elements were assigned between the steel loading plates and UHPFRC, between the steel support plates and UHPFRC, and between the steel web, including the continuous shear connectors, and UHPFRC. The same normal stiffness, tangential stiffness, cohesion, friction coefficient and tensile strength listed in Appendix E were used for all these contacts. A single parameter set was adopted as a simplified engineering assumption, without further contact-specific calibration. Interface opening was activated in the Interval Data tab, allowing relative slip and separation at these contacts.

**Figure 22:**
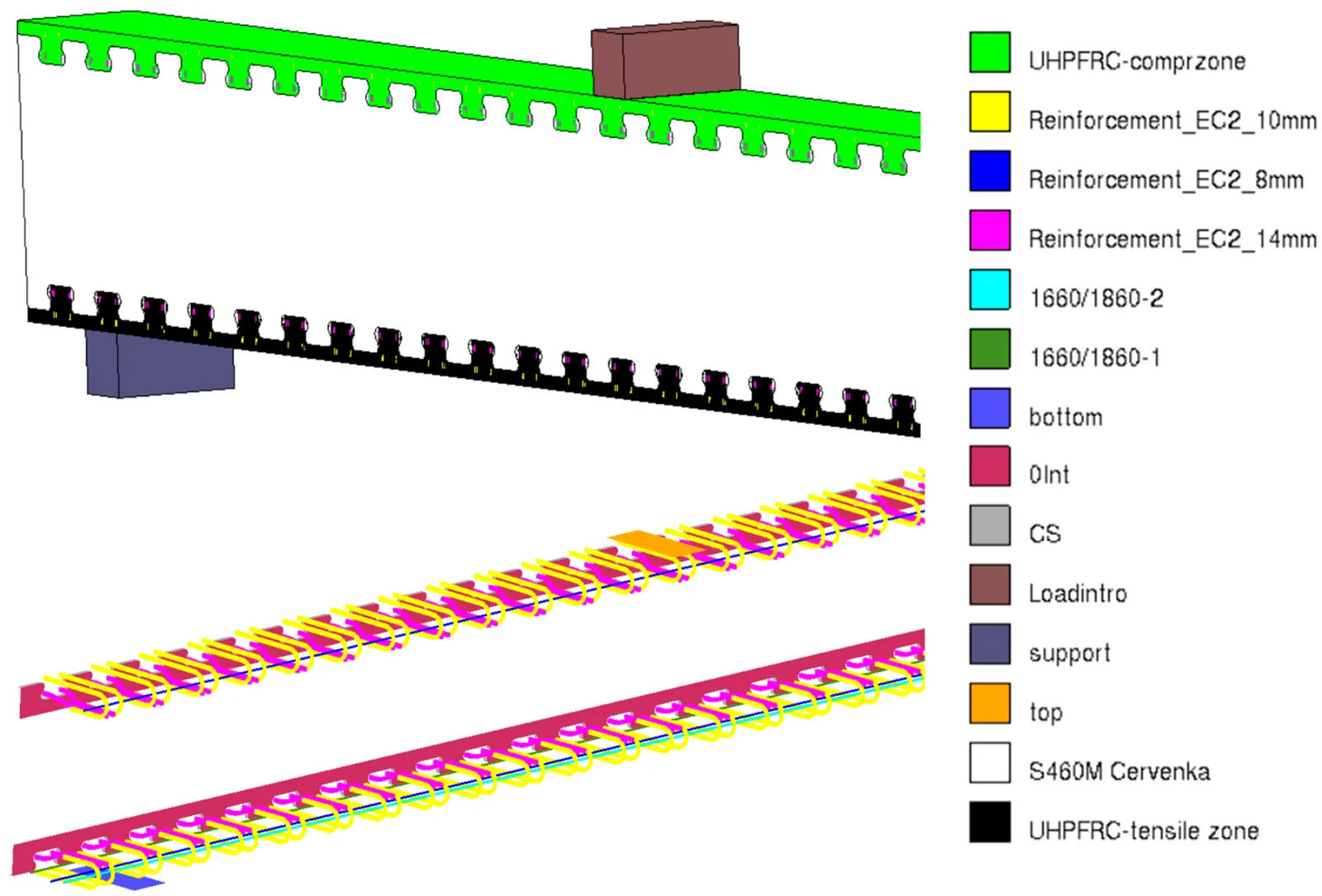
Material models (**right**); material models in FE model (**top left** and **bottom left**).

The internal reinforcement was modelled exclusively using 1D reinforcement elements, which were constrained along the symmetry plane to ensure correct symmetry behaviour. The prestressing was applied as a “prestressing for Reinf Line”, with the direction “Start Node” and a value of 1333 MPa. To correctly represent the prestressing behaviour, each individual strand was assigned a separate user-defined material model, as required by ATENA Science. This allowed the incorporation of the calibrated bond–slip relationships and the strand-specific stress–strain laws described in Section 3.1.2. Figure 22 gives an overview of all Material models in the final FE model. A separate UHPFRC model was assigned for the tensile and the compression flange, respectively. This allowed taking into account the slightly different mean compressive strengths measured experimentally for the tensile and compression flanges (152.7 and 154.7 N/mm^2^).

**Figure 23:**
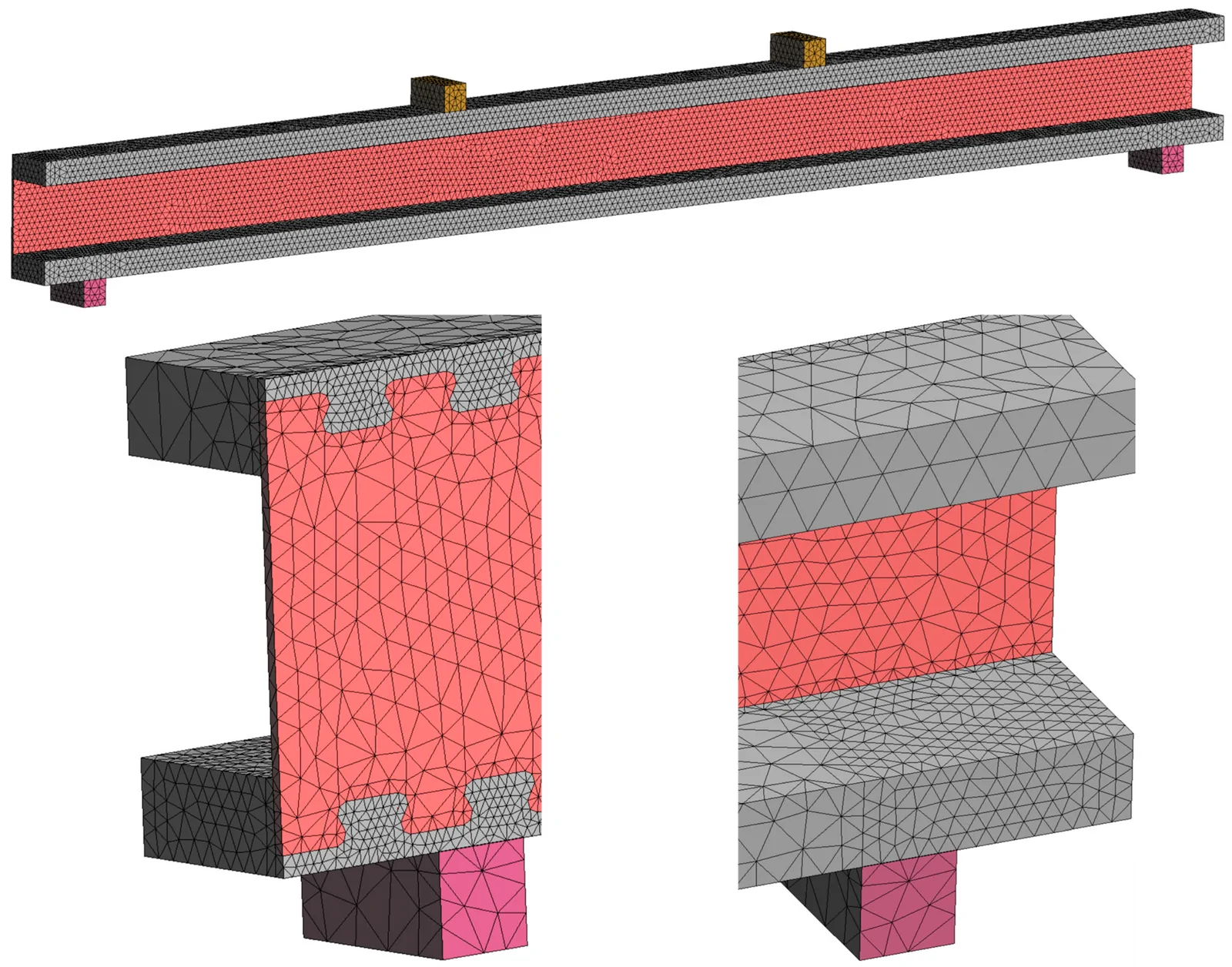
Final meshed FE model (**top**); preliminary locally refined mesh (subsequently discarded) (**bottom**).

The FE mesh was initially refined in all regions that are critical for the structural response. In particular, the mesh was locally densified from the global element size of 5 cm down to 1 cm, specifically around the shear connectors, in the anchorage zone of the prestressing strands in the lower UHPFRC flange, and along the steel web. This refinement was introduced with the intention to reduce artificial stress concentrations and more accurately capture the local stress gradients in these highly nonlinear regions (Figure 23).

Initial simulations with locally refined meshes showed reduced deformation capacity for the adopted user-defined material inputs. The original comparisons coupled changes in discretisation with changes in characteristic-size settings and therefore did not isolate a mesh effect. A reduction in dissipated fracture energy caused solely by smaller integration volumes was not demonstrated and is not inferred here from the crack-band concept [53]. Subsequently performed analyses use uniform nominal tetrahedral mesh sizes, and separate sensitivity studies distinguish the tensile material-reference-size influence from the discretisation influence (Section 4.1). A hexahedral mesh could not be generated consistently across the complex interfaces and connector geometry. The 35 mm reference mesh of the fully reinforced four-point-bending model I-2 contains 7346 1D elements, 120,408 tetrahedral elements and 46,724 nodes.

## 4. FE Model Results

### 4.1. Mesh Sensitivity and Reference Discretisation—Beam I-2

Beam I-2 served as the structural reference for model selection and sensitivity assessment. The material-curve shapes identified in Section 3.1 were retained, but the Tension Characteristic Size was assessed against the I-2 response. Two separate comparisons were performed: Tension Characteristic Size values of 25, 35 and 50 mm at a fixed 35 mm mesh, and nominal mesh sizes of 25, 35 and 50 mm at a fixed Tension Characteristic Size of 25 mm. The tensile reference-size setting was changed consistently in both UHPFRC flange materials. All other inputs were held at the reference-model values in each comparison, including the compression reference size, structural-steel material, bond law, interfaces and localisation-onset setting. The resulting selection on I-2 is distinguished from subsequent transfer to I-1 and I-3.

**Figure 24:**
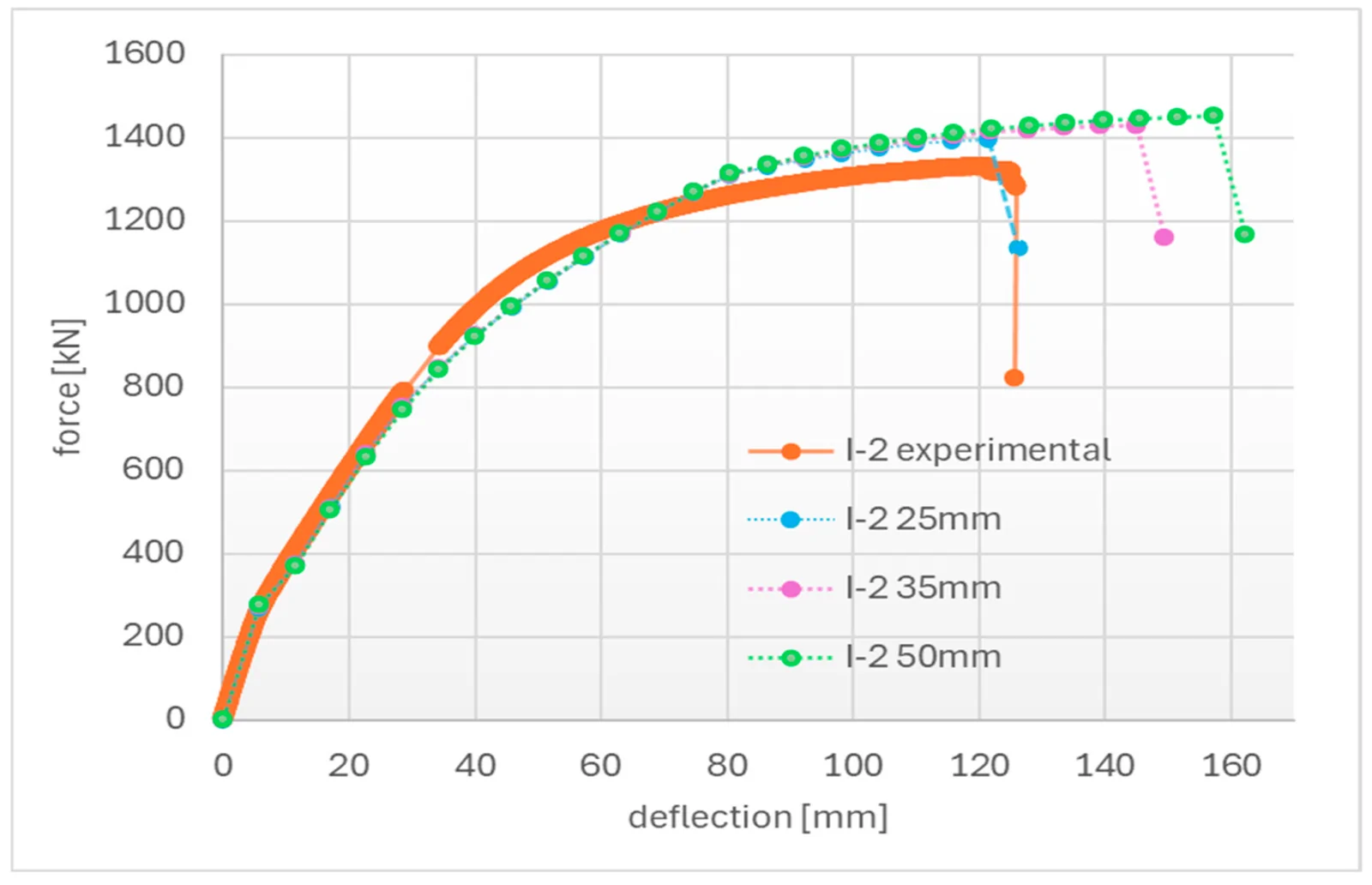
Sensitivity to UHPFRC Tension Characteristic Size (25, 35 and 50 mm) at a fixed 35 mm mesh; all other inputs unchanged.

Figure 24 shows the influence of Tension Characteristic Size at a fixed 35 mm mesh. Increasing this input from 25 to 35 and 50 mm increased the peak load from 1396.0 to 1428.9 and 1452.0 kN, respectively, while the corresponding peak-load deflection increased from 121.5 to 144.9 and 157.3 mm. Across these alternatives, the peak-load range is 4.0%, and the peak-load-deflection range is 29.5%, each relative to the smallest value. All cases reached the strand-rupture criterion. The main sensitivity concerns the predicted deformation at the ultimate stage, while substantial portions of the preceding load–deflection paths remain similar. The 25 mm input was selected because it gave the closest agreement with the I-2 peak-load deflection among the investigated alternatives. This is an additional member-level calibration choice; it does not change the inverse-analysis conversion length of 37.5 mm and does not establish equivalence between those two quantities.

**Figure 25:**
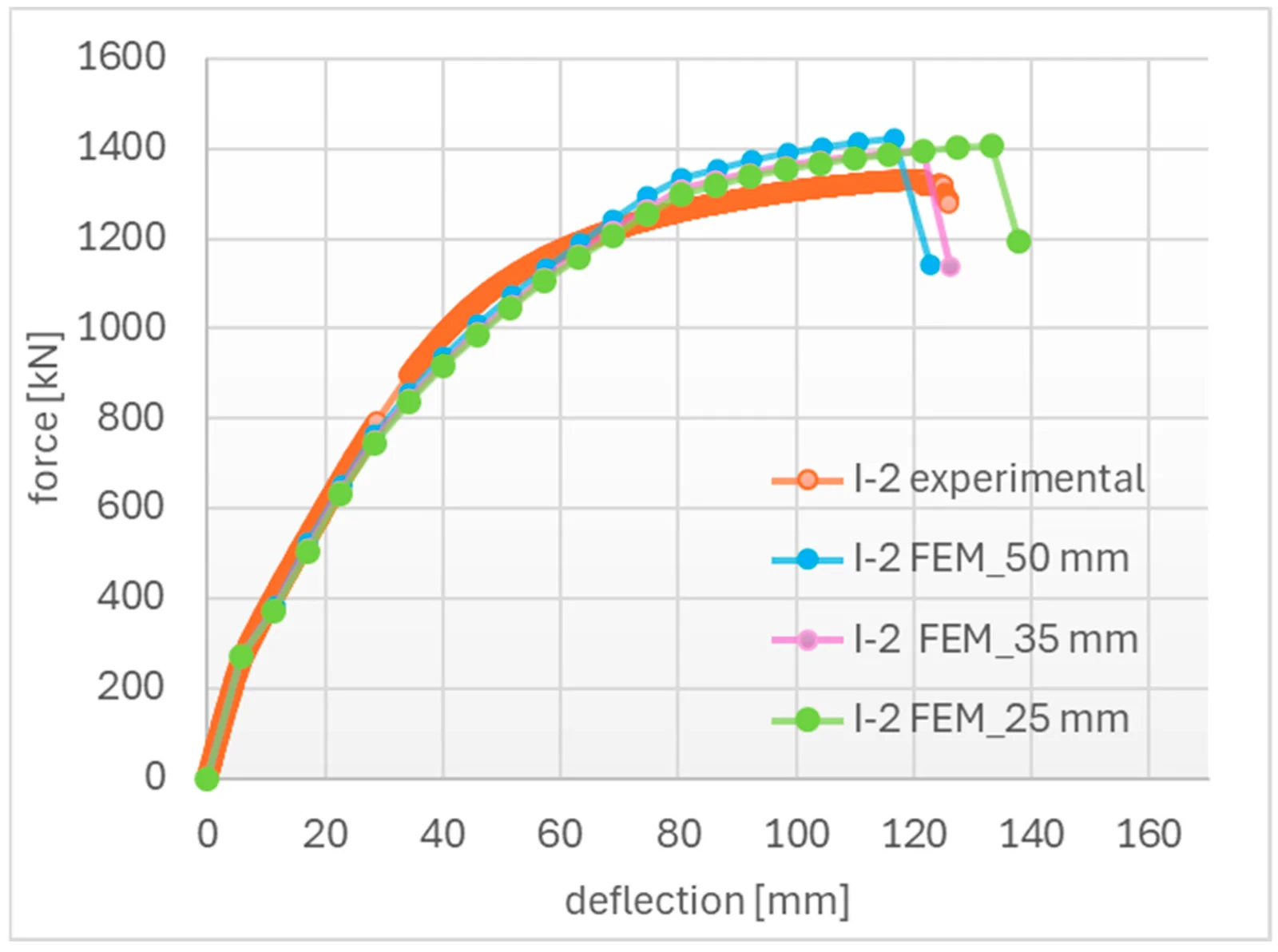
Mesh sensitivity of I-2 with Tension Characteristic Size fixed at 25 mm; nominal mesh sizes are 50, 35 and 25 mm.

Figure 25 compares the three mesh sizes at a constant Tension Characteristic Size of 25 mm. The peak loads are 1422.3, 1396.0 and 1407.1 kN for the 50, 35 and 25 mm meshes, respectively, a range of 1.9% relative to the smallest value. The corresponding peak-load deflections are 116.6, 121.5 and 133.3 mm, a range of 14.3%. All three discretisations reached the strand-rupture criterion. Thus, the peak load is comparatively insensitive to mesh size within the investigated range, while appreciable deformation sensitivity remains.

Table 4 reports the mesh-study results using peak load and its corresponding centre deflection as separate, consistently defined metrics. The experimental reference curve reaches 1331.0 kN at 120.2 mm; its largest recorded pre-drop deflection of approximately 126 mm is a different quantity. The 35 mm mesh is retained as a practical reference discretisation: it gives a response close to those of the other meshes and the closest peak-load deflection to I-2 among the three discretisations. These results do not establish complete mesh objectivity or convergence of the terminal deformation.

The common strand-controlled failure mechanism is retained across the investigated mesh sizes, but the remaining deformation sensitivity must be considered when interpreting localised failure and post-peak increments. The selected 35 mm mesh and 25 mm Tension Characteristic Size were then used unchanged for I-1, I-3 and the additional I-4 challenge case. No further specimen-related tuning was performed.

### 4.2. Cross-Specimen Structural Validation—Beams I-1 to I-3

Following selection of the 35 mm reference mesh and 25 mm Tension Characteristic Size using I-2, the same modelling strategy was applied to beams I-1 and I-3 without recalibration of the constituent material models. The 35 mm mesh size, 25 mm tensile material-reference size, UHPFRC constitutive relationships, structural-steel and prestressing-strand material laws, bond–slip formulation and interface parameters were kept unchanged. Only the physical differences between the experimental specimens were reproduced in the respective FE models. Beam I-1 was modelled according to the three-point bending configuration with its complete reinforcement layout, whereas beam I-3 retained the four-point bending configuration of I-2 but without the additional dowel reinforcement. Consequently, beams I-1 and I-3 constitute predictive structural validation cases: neither specimen was used to identify constituent material laws, bond parameters or the reference numerical settings. I-4 was additionally analysed with the same selected inputs, changing only its specimen-specific reinforcement configuration.

**Figure 26:**
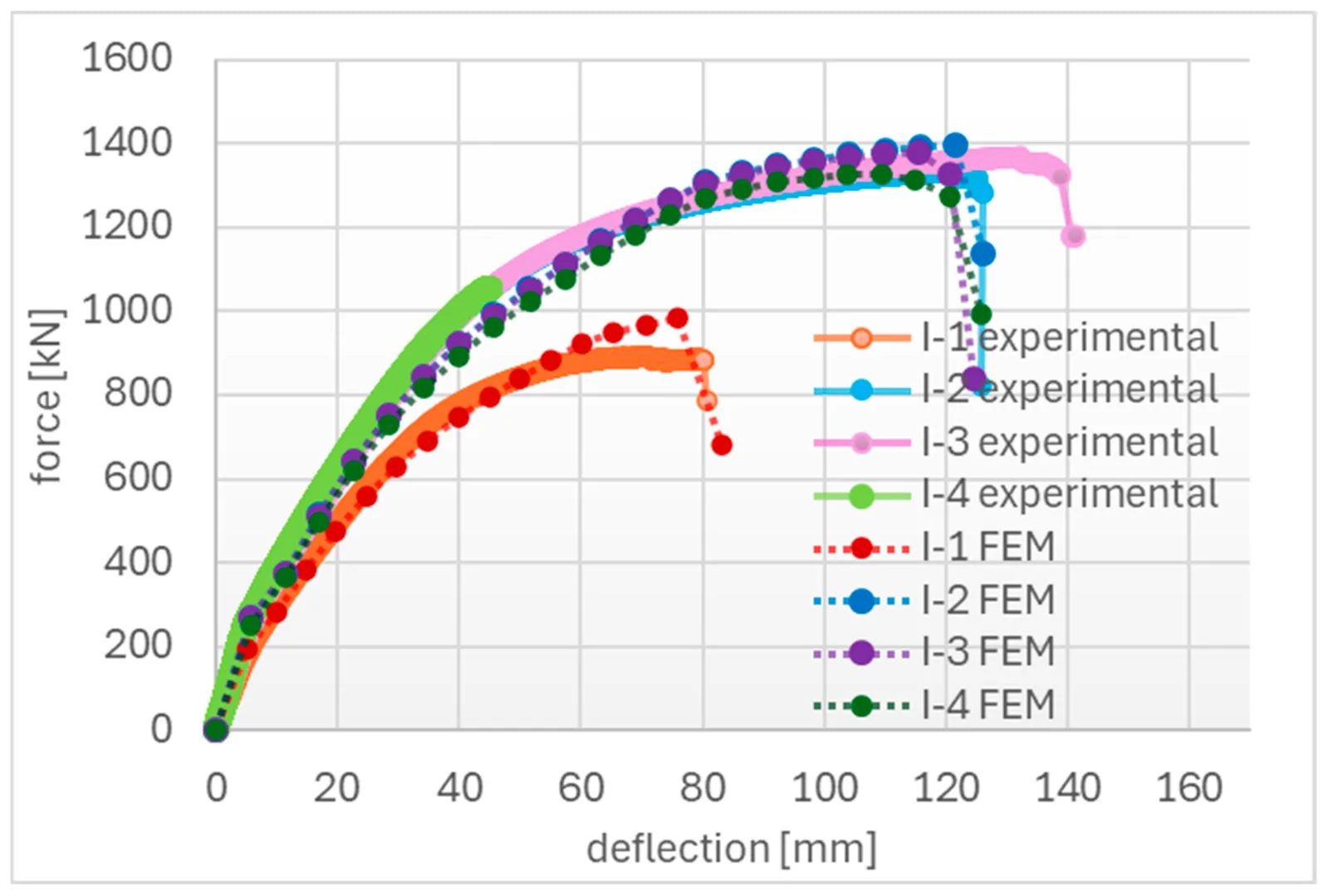
Experimental and numerical load–deflection curves of I-1 to I-4 with the selected 35 mm mesh and 25 mm Tension Characteristic Size. I-4 is a boundary-of-validity case. Solid curves represent experiments; dotted curves represent FE results (I-1 red, I-2 blue, I-3 purple and I-4 dark green).

Figure 26 compares the experimental and numerical load–deflection curves of I-1 to I-4. The selected model reproduces the lower capacity of the three-point-bending specimen I-1 and the higher, similar capacities of the four-point-bending specimens I-2 and I-3. The numerical peaks for I-1, I-2 and I-3 are 982.6, 1396.0 and 1377.3 kN at 75.9, 121.5 and 115.3 mm, respectively. Differences in the highly nonlinear range are assessed separately for peak load and peak-load deflection. I-4 is shown to document the limitation for a different experimental failure mechanism; it is not included in the detailed strand-controlled field comparison.

Using the paired peak-load values from the plotted experimental curves (Table 5), the numerical peak-load deviations for I-1, I-2 and I-3 are +10.1%, +4.9% and +0.7%, respectively. The corresponding deflection deviations are +8.3%, +1.0% and −12.7%. These deflections are evaluated at each curve’s own load maximum and are not the maximum recorded displacements or displacements at the final load drop. The numerical peak loads are consistently higher than the experimental values, while the peak-load deflections are overpredicted for I-1 and I-2 and underpredicted for I-3. The available comparisons do not isolate the individual contributions of material scatter, prestress losses and other modelling assumptions.

The I-4 simulation reached a maximum of 1324.1 kN at 109.5 mm, followed by a descending branch and attainment of the strand-rupture criterion. The experimental curve reaches 1056.0 kN at approximately 45.0 mm and terminates through longitudinal splitting of the tensile flange. The model therefore overpredicts the experimental peak load by 25.4% and does not reproduce the governing failure mechanism. The longitudinal symmetry constraint restricts development of the observed splitting mechanism. I-4 is consequently shown as a boundary-of-validity case in Figure 26 and is excluded from the subsequent constituent-field analysis; the agreement metrics for strand-controlled specimens refer only to I-1 to I-3.

Particularly relevant is the transfer of the modelling strategy between the different structural configurations. The model correctly reproduces the pronounced reduction in load-bearing capacity associated with the three-point bending configuration of I-1, while the four-point bending specimens I-2 and I-3 reach similar ultimate load levels despite the absence of dowel reinforcement in I-3. This relative behaviour is consistent with the experimental campaign. The agreement therefore extends beyond the reproduction of a single reference test and supports transfer of the selected model settings to the additional strand-controlled specimens when the experimentally defined loading and reinforcement configurations are changed.

While the global response provides an overall assessment of the predictive capability of the modelling strategy, it does not demonstrate whether the experimentally observed internal failure mechanisms are reproduced correctly. The following section therefore evaluates the stress, strain and displacement fields of the individual FE models and compares the numerically obtained failure sequence with the experimental observations.

### 4.3. Governing Failure Mechanisms and Comparison with Experimental Observations

#### 4.3.1. Prestressing-Strand Response and Failure

The experimental campaign showed that beams I-1 to I-3 ultimately failed by rupture of at least one prestressing strand, despite the differences in loading configuration and reinforcement layout. Reproduction of this governing failure mode therefore provides an important local validation criterion in addition to the global load–deflection response. The stress and strain states of the prestressing strands were consequently evaluated at the respective maximum load Fmax and in the first converged calculation step following the peak load. The peak-load state, the first converged post-peak state and the ultimate strand-failure state are distinguished: they need not coincide, as the I-3 load maximum precedes its later major load drop.

**Figure 27:**
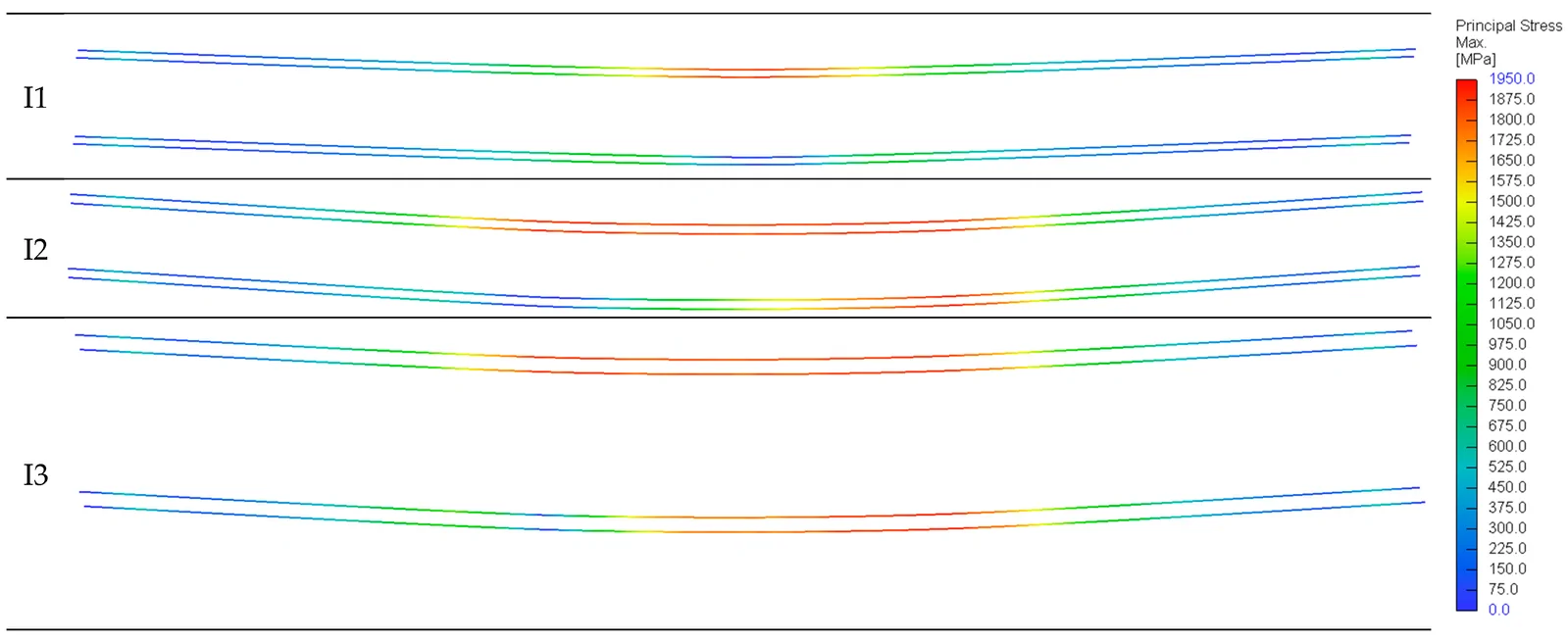
Axial strand stress distribution in the prestressing strands of beams I-1 to I-3 at Fmax (**top**) and in the first converged post-peak calculation step (**bottom**); identical stress scale of 0–1950 MPa.

Figure 27 shows that the spatial distribution of strand stresses is directly related to the bending-moment distribution of the respective test configuration. In the three-point bending specimen I-1, the highest strand stresses are concentrated around the central maximum-moment region. In contrast, beams I-2 and I-3 exhibit an extended zone of nearly uniform high strand stress within the constant-moment region between the two load-introduction points. In all three simulations, the strands are mobilised into the ultimate stress range at Fmax. In the first converged post-peak step, local reduction and redistribution of strand stress can be assessed. The identification of strand rupture additionally requires checking the corresponding strain-based criterion at the relevant increment. The ultimate strand-controlled loss of capacity is reflected by the later global load drop; its timing must not be inferred solely from a field labelled as the first post-peak state. All prestressing strands reach stresses close to their ultimate load-bearing capacity of 1920 N/mm^2^.

**Figure 28:**
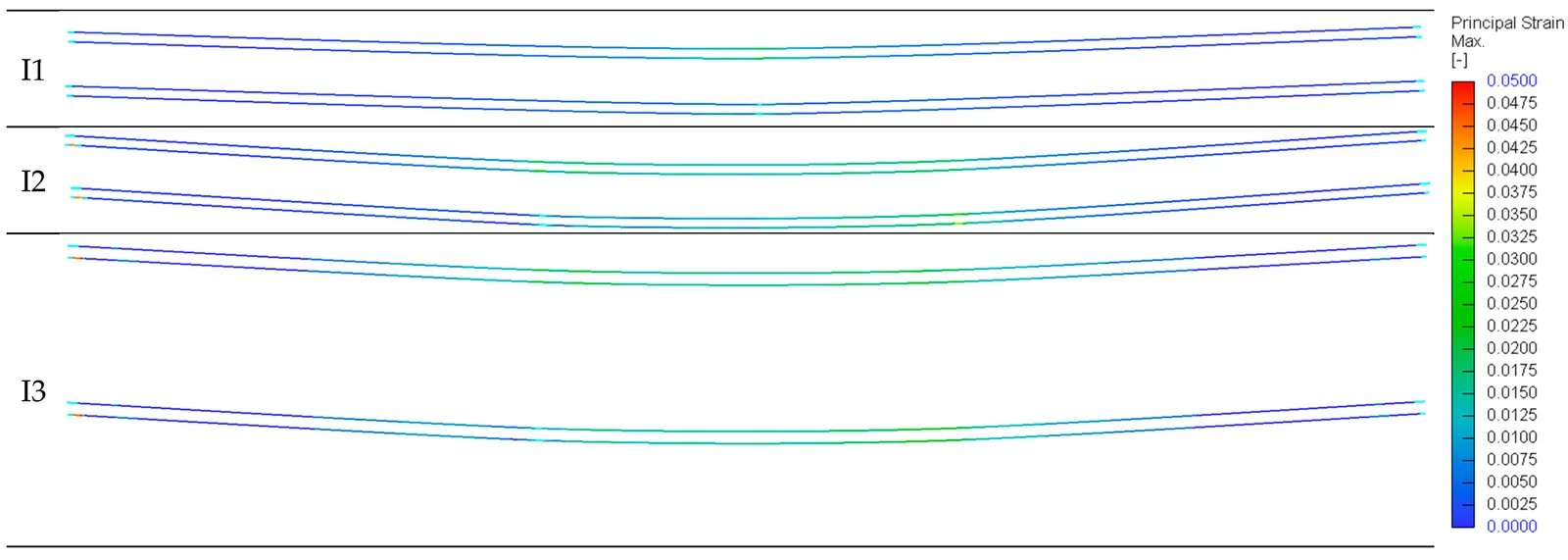
Axial strand strain distribution in the prestressing strands of beams I-1 to I-3 at F_max_ (**top**) and in the first converged post-peak calculation step (**bottom**). The constitutive ultimate strain of the strand model is 4%; bright-cyan regions indicate values exceeding the displayed upper contour limit of 5%.

The corresponding strain fields (Figure 28) complement the strand-stress evaluation. The spatial distributions reflect the concentrated maximum-moment region of I-1 and the extended constant-moment regions of I-2 and I-3. Localised strain concentrations develop during the nonlinear response. Where the constitutive ultimate strain of 4% is exceeded, the field indicates that the adopted strain-based limit has been reached; values above the displayed contour limit are not physical predictions of post-rupture strand strain. The peak-load and first post-peak fields alone do not establish an identical sequence of threshold exceedance for all three specimens.

The stress and strain fields should be interpreted together with the load–deflection history and the attained strand-failure criterion. The analyses of I-1 to I-3 reach strand-controlled ultimate failure, consistent with the experimental failure mode. This agreement concerns the governing failure mechanism; it does not require the strand criterion to coincide exactly with the global load maximum. Large local strains after the criterion is exceeded represent the constitutive/numerical response and redistribution, not a calibrated physical fracture strain after rupture.

Particularly relevant is that the location and spatial extent of the critical strand response changes consistently with the loading configuration while the same strand material and bond laws are retained. The concentrated response of I-1 and the extended high-stress region of I-2 and I-3 demonstrate that the numerical failure pattern is governed by the structural moment distribution rather than by a prescribed or locally calibrated failure location. Moreover, the absence of dowel reinforcement in I-3 does not alter the governing ultimate failure mode, consistent with the experimental observation that I-3 also reached failure by strand rupture.

#### 4.3.2. Steel-Web Response and Inter-Dowel Web Yielding

The response of the 20 mm thick S460 ML steel web was evaluated to investigate the development and redistribution of local plastic deformation between the continuous puzzle-shaped shear connectors. Inter-dowel web yielding (IWY) had previously been identified in the experimental campaign [25] as a characteristic mechanism in the tensile region of the web. In the reference simulation of I-2, the experimentally determined yield strength of the S460 ML steel was reached locally between approximately 510 and 640 kN, confirming that the observed strain concentrations correspond to yielding of the steel web rather than to a purely elastic strain localisation.

**Figure 29:**
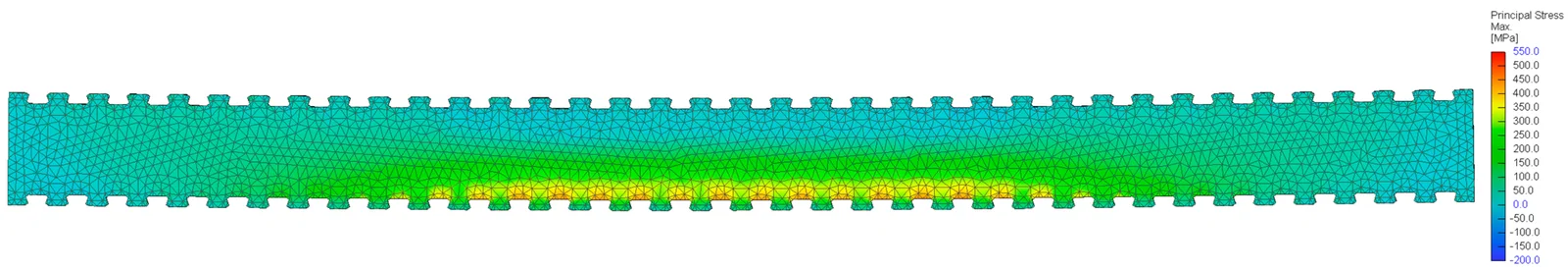
Stress distribution in the S460 ML steel web of beam I-2 during the onset of inter-dowel web yielding at approximately 640 kN, displayed stress range −200 to +550 N/mm^2^.

As illustrated in Figure 29, yielding develops locally in the tensile region of the steel web between adjacent puzzle-shaped connectors. This stress-based evaluation provides the basis for using the principal-strain fields below to assess the spatial development of IWY throughout the highly nonlinear response.

**Figure 30:**
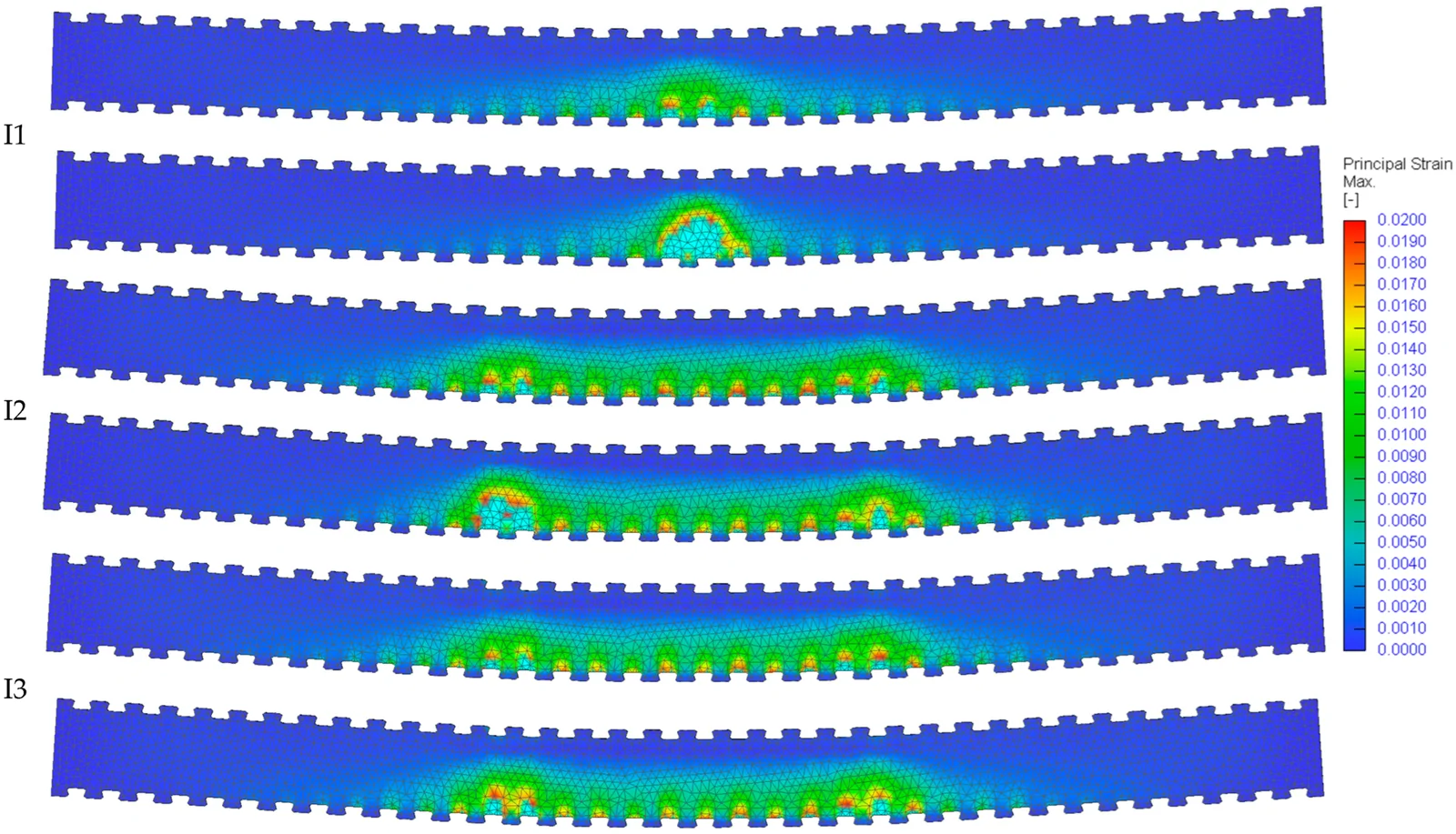
Maximum principal strain distribution in the isolated S460 ML steel webs of beams I-1 to I-3 at F_max_ (**top**) and in the first converged post-peak calculation step (**bottom**); identical strain scale of 0–2%.

The principal-strain distributions at F_max_ in the S460 ML steel web (Figure 30) show pronounced localisation in the tensile region of the steel web between adjacent puzzle-shaped connectors for all three specimens. The spatial extent of this yielding pattern is strongly influenced by the bending-moment distribution. In the three-point bending specimen I-1, the highly strained web region remains concentrated around the central maximum-moment zone. In contrast, the four-point bending specimens I-2 and I-3 develop a substantially more extended region of high web strains of around 20,000 µm/m and more (cyan coloured) within the constant-moment zone between the load-introduction points. Within these regions, repeated local strain concentrations occur between successive dowel sections, reproducing the characteristic spatial pattern associated with inter-dowel web yielding.

The first converged calculation step following Fmax reveals a pronounced expansion of the highly strained web regions in all three specimens. These fields document redistribution after the global peak; the timing relative to local strand-failure-criterion exceedance must be evaluated separately for each specimen. The numerical sequence therefore indicates a redistribution of internal forces during the post-peak response, with an increased contribution of the yielding steel web to the remaining load-transfer mechanism. Importantly, web yielding is already present before strand failure; the post-peak response represents an expansion of the existing IWY zones rather than the initiation of a new failure mechanism.

The web response therefore demonstrates two complementary aspects of the modelling strategy: the experimentally identified IWY mechanism [25] is reproduced locally between adjacent connectors, while its spatial development responds consistently to the different global moment distributions of the three specimens. The experimentally measured strain fields used for an independent spatial comparison with these numerical results are discussed separately in Section 4.3.4.

#### 4.3.3. Local UHPFRC Response and Connector Mechanisms

While the response of the prestressing strands and the steel web can be related directly to experimentally observed failure modes and surface strain measurements, the internal stress state of the UHPFRC surrounding the continuous shear connectors is not accessible with comparable spatial resolution in the large-scale tests. The three-dimensional FE model is therefore used to interpret the internal load-transfer mechanisms numerically, rather than to claim direct experimental validation of these internal states. For this purpose, the principal-stress distribution, the development of compressive inelasticity and the transverse displacement field of the UHPFRC were evaluated separately.

**Figure 31:**
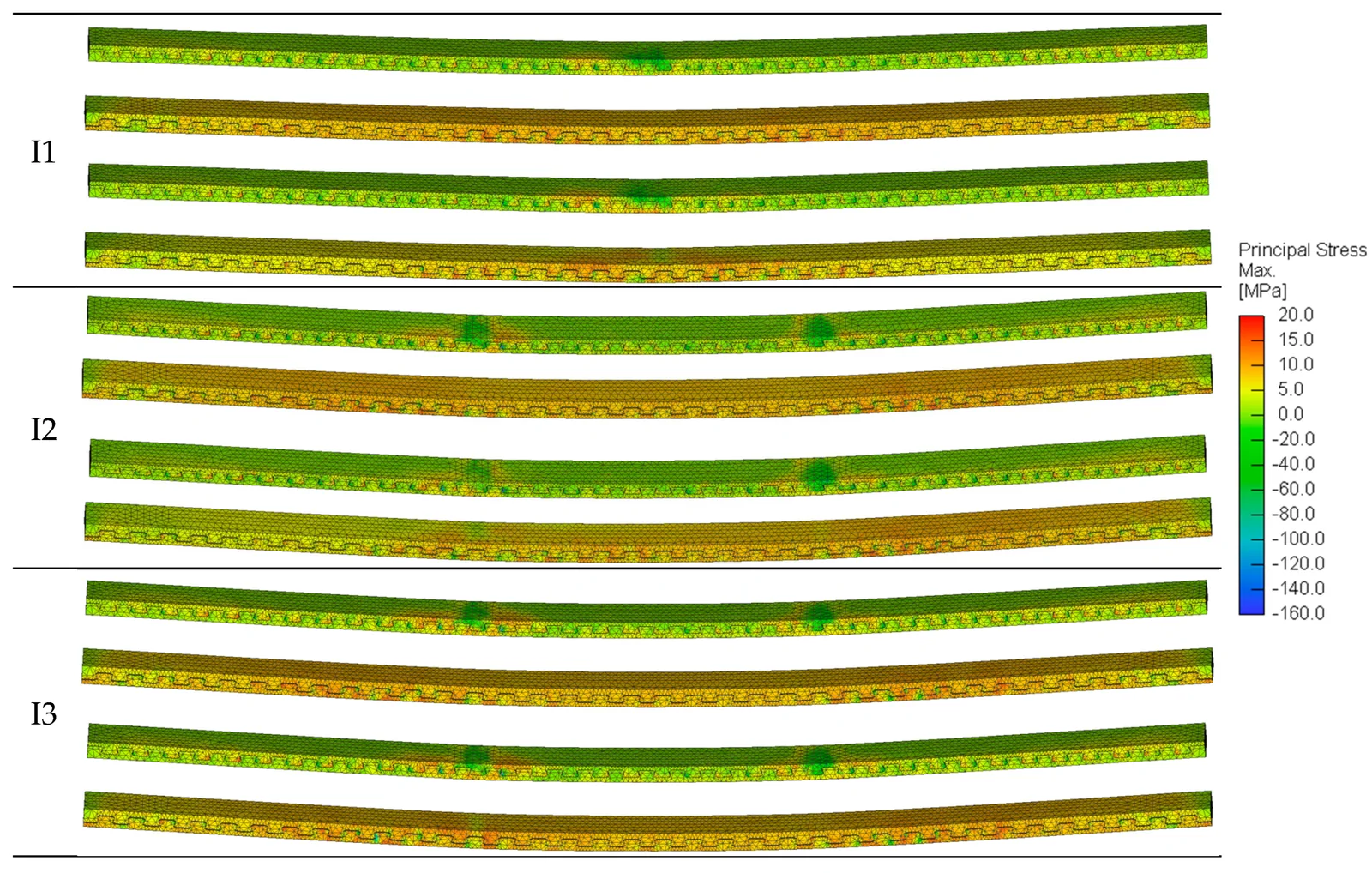
Maximum principal-stress distribution in the isolated UHPFRC flanges of beams I-1 to I-3 at F_max_ (**top**) and in the first converged post-peak calculation step (**bottom**); identical stress scale for all specimens.

The maximum principal-stress fields in Figure 31 were evaluated primarily to identify tensile stress concentrations and the resulting splitting-sensitive stress state within the UHPFRC flanges. Pronounced local tensile stress concentrations occur in the vicinity of the puzzle-shaped connectors, particularly within the tensile flange, where the global flexural tensile state interacts with the local transfer of forces between the steel web and the UHPFRC. Since the displayed quantity represents the maximum principal stress, it is not used to quantify the governing compressive stress in regions subjected to multiaxial compression; the compressive nonlinear response is evaluated separately below using the material-state indicator.

The overall UHPFRC stress patterns of I-2 and I-3 remain qualitatively similar despite the absence of the additional dowel reinforcement in I-3. This indicates that the fundamental connector-related load-transfer mechanism is retained in both four-point bending specimens and is consistent with their comparable global capacities and common strand-controlled ultimate failure.

Following local strand failure, the stress state of the tensile flange changes markedly, with pronounced local unloading and redistribution in the connector region. This response is consistent with the simultaneous reduction in strand stress and expansion of steel-web yielding described in Section 4.3.1 and Section 4.3.2 and demonstrates that the post-peak response involves redistribution between all principal constituents of the composite cross-section.

**Figure 32:**
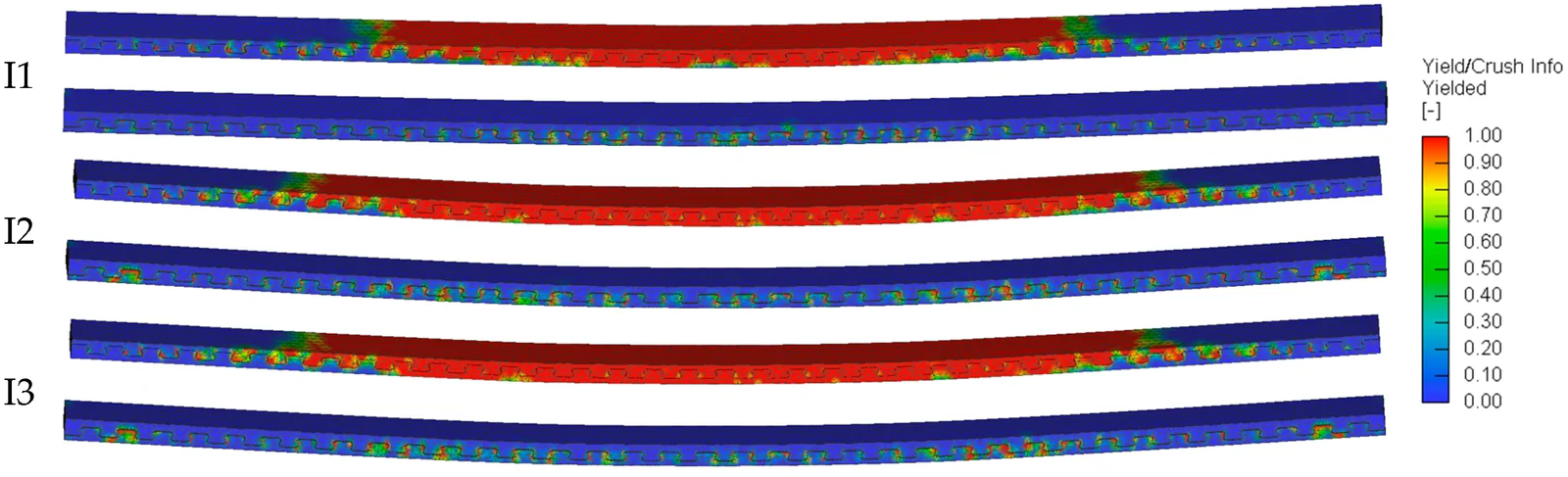
Compressive yield-state indicator (“Yield/Crush Info—Yielded”) of the UHPFRC in beams I-1 to I-3 at F_max_; values approaching 1 identify regions in which the compressive plasticity criterion has been activated.

The material-state indicator in Figure 32 complements the principal-stress evaluation by identifying regions in which the nonlinear compressive response of the UHPFRC has been activated. At Fmax, extensive portions of the upper compression flange have entered the non-elastic state in all three beams, indicating substantial mobilisation of the UHPFRC compression zone within the adopted constitutive model in line with the assumption made in [25] that at ULS the complete cross section of the compression flange can be utilised. In the tensile flange, compressive yielding remains predominantly localised around the continuous connectors, consistent with the highly concentrated multiaxial load-transfer state in these regions. The displayed “Yielded” state indicates activation of compressive plasticity but does not, by itself, imply that the material has already entered the descending softening branch or undergone complete crushing.

This distinction is consistent with the experimental failure sequence. While substantial compressive inelasticity may develop before the maximum member load is reached, the compression flange did not govern the ultimate failure of beams I-1 to I-3. Following strand rupture, continued deformation and redistribution of internal forces can further increase local compressive strains and eventually lead to the crushing observed experimentally. The present results therefore support progressive mobilisation of the UHPFRC compression zone prior to strand-controlled failure without implying premature global compression failure.

**Figure 33:**
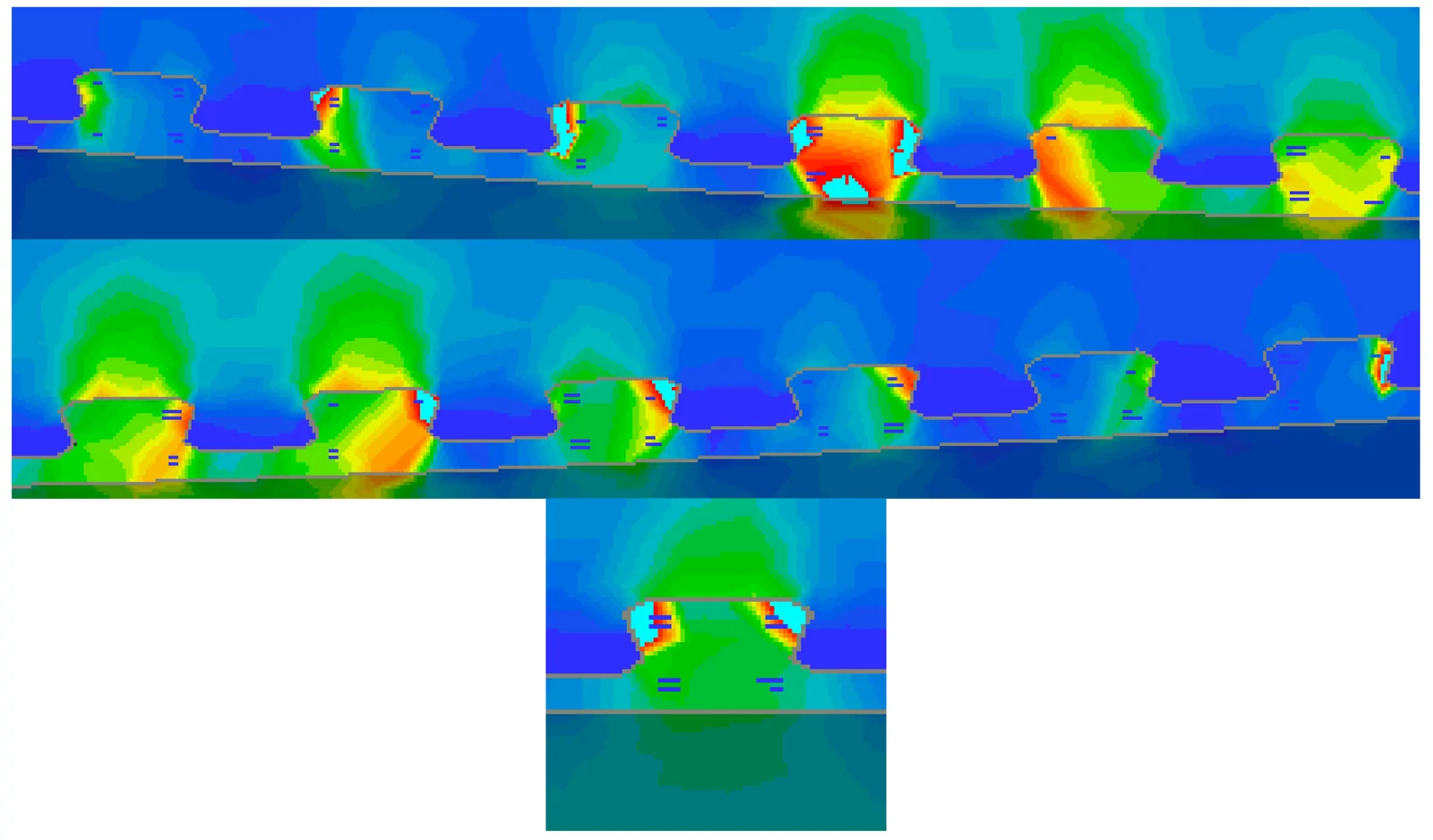
I-2 connector-level case study at F_max_: connector regions on the left beam side (**top**), right beam side (**middle**) and at midspan (**bottom**), illustrating the localised deformation state around the connector roots.

While Figure 32 identifies the spatial extent of compressive plasticity on member level, a detailed evaluation of beam I-2 provides further insight into the local interaction between the continuous connector and the surrounding UHPFRC. Figure 33 therefore shows representative connector regions at different longitudinal positions of the tensile flange.

The detailed fields reveal pronounced localisation of the nonlinear UHPFRC response at the connector roots and along the connector–concrete contact region. Although the lower flange is subjected globally to flexural tension, the transfer of shear forces through the continuous connector, combined with the relative rotation of the steel web and UHPFRC flange caused by beam curvature, generates local compressive actions within the surrounding concrete. Consequently, tensile splitting demand and local compressive inelasticity coexist within a comparatively small connector region.

The orientation of the localised response changes along the beam length in accordance with the relative rotation between the steel web and the UHPFRC flange. On opposite sides of the beam, the predominant contact region therefore occurs on opposite sides of the puzzle-shaped connector, whereas the connector region close to midspan exhibits a more symmetric local response. This behaviour illustrates that the concrete surrounding the connector is subjected to a three-dimensional stress state that cannot be inferred from the global bending stress distribution alone. The local stress and material-state response described above is accompanied by a numerically evaluated kinematic response of the flange. To examine this aspect, the transverse displacement of the beams was evaluated.

**Figure 34:**
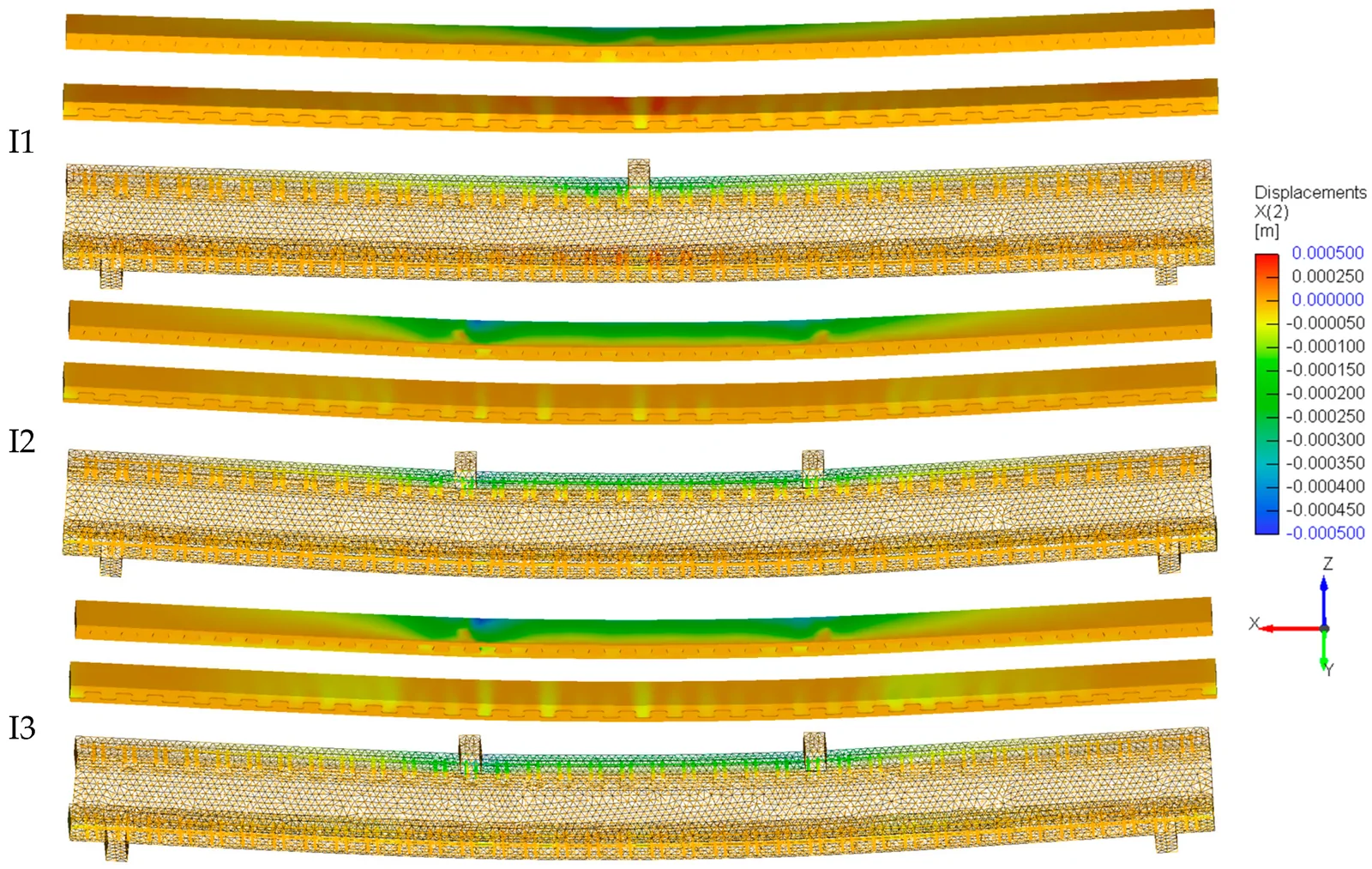
Transverse displacement u_2_, perpendicular to the longitudinal beam axis, at F_max_ for beams I-1 to I-3. Isolated UHPFRC flanges (**top**) and corresponding wireframe representations including the embedded reinforcement (**bottom**); identical displacement scale for all specimens. Positive and negative values represent opposite transverse displacement directions.

A complementary view of the connector mechanism is obtained from the transverse displacement component u_2_ (y-direction, see Figure 34), oriented perpendicular to the longitudinal beam axis. The isolated UHPFRC plots reveal local lateral displacement of the tensile flange in the regions surrounding the continuous connectors. The opposite displacement directions across the flange represent the kinematic manifestation of a transverse opening tendency and are therefore consistent with the splitting-sensitive stress state identified from the principal-stress fields.

In beam I-1, this transverse deformation remains predominantly concentrated in the central highly loaded load-introduction region, whereas I-2 and I-3 exhibit a more distributed lateral deformation pattern over the highly stressed four-point bending region. The correspondence between the global moment distribution, the internal UHPFRC stress field and the resulting transverse deformation provides a mechanically consistent picture of the splitting tendency within the tensile flange. The upper compression flange exhibits a distinct transverse displacement pattern associated with the progressive deformation of the compressed UHPFRC and the overall cross-sectional response.

The wireframe representations show the embedded conventional reinforcement within the regions affected by transverse UHPFRC deformation. The spatial correspondence illustrates its potential restraint of lateral opening and is consistent with the experimentally observed importance of reinforcement placement for controlling splitting deformations. No quantitative reinforcement force is inferred from the displacement field alone.

Taken together, the principal-stress fields, the compressive yielding/crushing indicator and the transverse displacement results provide a three-dimensional description of the UHPFRC connector response that could not be obtained directly from the large-scale experiments. The simulations identify local tensile splitting demand, localised compressive inelasticity and the associated transverse deformation within the same connector regions, while the governing member failure remains strand-controlled. These internal fields are numerical interpretations of mechanisms inferred from the preceding experimental campaigns [23,25]; they were not directly measured in the large-scale beam tests.

#### 4.3.4. Qualitative Comparison with DIC Strain Fields

Digital image correlation measurements from the large-scale beam tests provide an independent full-field reference for assessing whether the spatial strain distributions predicted by the FE models correspond to the experimentally observed structural response. In contrast to the preceding global load–deflection comparison, the DIC data support a qualitative assessment of the location and spatial extent of strain localisation within the visible steel web. The comparison was therefore performed for beams I-1 to I-3 using an identical displayed strain range of 0–2% for the numerical and experimental results.

The comparison assesses qualitative spatial strain-localisation patterns using an identical contour range. For each beam, the experimental and numerical fields are shown at their respective peak-load states rather than at an identical absolute load. The comparison therefore concerns the location and spatial extent of localisation, not pointwise agreement of strain magnitudes.

**Figure 35:**
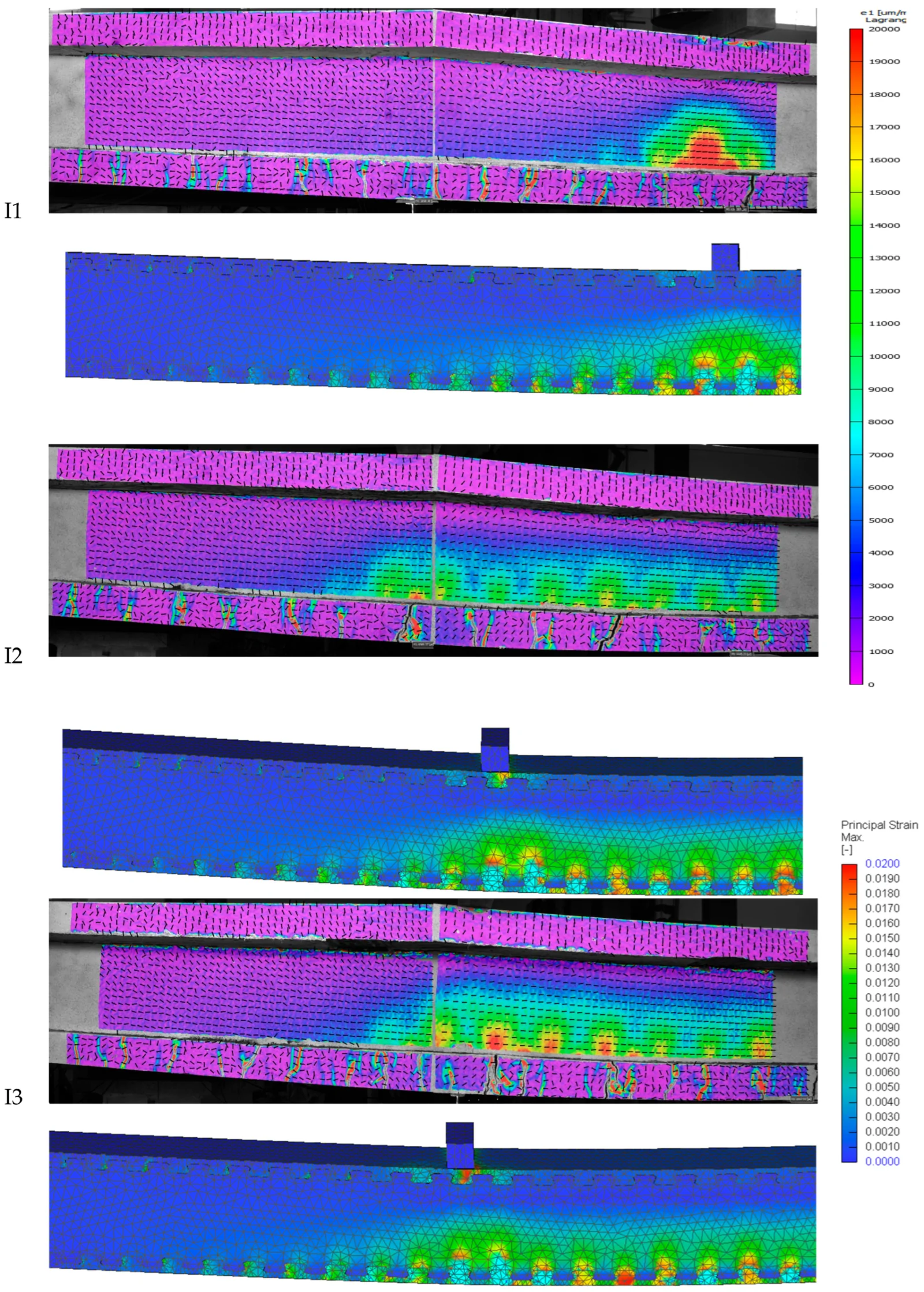
Comparison of the DIC major-principal-strain fields experimentally measured (**top**) and the numerically predicted maximum principal strain fields (**bottom**) for beams I-1 to I-3 close to Fmax; identical displayed strain range of 0–2%. The DIC field is limited to the experimentally visible side of the beam; Experimental DIC images reproduced from Harsányi et al. [25], under CC BY 4.0 https://creativecommons.org/licenses/by/4.0/ (accessed on 28 October 2026); numerical fields prepared for this study.

The comparison in Figure 35 shows qualitative spatial correspondence in the location and spatial extent of the highly strained regions. For the three-point bending specimen I-1, both experiment and simulation exhibit a comparatively concentrated high-strain zone around the central maximum-moment region. In contrast, the four-point bending specimens I-2 and I-3 develop an extended highly strained region in the lower part of the steel web within the constant-moment zone. The FE models therefore reproduce not only the difference in global load-bearing behaviour between the loading configurations, but also the corresponding change from a concentrated to a distributed strain-localisation pattern.

Within these highly strained regions, the numerical and experimental fields further exhibit repeated local concentrations between adjacent puzzle-shaped connectors. This spatial correspondence provides independent qualitative evidence for the location of the inter-dowel web-yielding regions discussed in Section 4.3.2; it does not establish pointwise agreement of the strain magnitudes.

Particularly noteworthy is the similarity between I-2 and I-3. Despite the absence of the additional dowel reinforcement in I-3, the experimentally measured and numerically predicted strain fields show the same fundamental distributed web-yielding pattern. This finding is consistent with the comparable ultimate loads and common strand-controlled failure mode of the two specimens and supports the conclusion that omission of the dowel reinforcement did not fundamentally alter the governing load-transfer mechanism in these two configurations.

The qualitative spatial correspondence is relevant because the structural DIC fields were not used as calibration targets. Together with the global response and the strand-controlled ultimate failure, the contour comparison provides complementary support for the model’s representation of the observable behaviour of I-1 to I-3. It does not establish quantitative agreement of the full strain field, and it does not validate the internal UHPFRC stress state.

The global response, ultimate failure mode and qualitative surface-strain correspondence support using the models to interpret the internal fields in Section 4.3.3. These internal numerical interpretations remain complementary to the experimental evidence and are not independently validated by the DIC comparison.

## 5. Scope of Validity and Limitations

The modelling strategy presented above is subject to a number of limitations that define its range of applicability.

Validation basis: I-2 was used for structural model selection, including the compression-law alternative, mesh and Tension Characteristic Size, and is therefore not a purely independent validation specimen. I-1 and I-3 were analysed subsequently without further specimen-specific adjustment and provide transfer-assessment cases for a different loading arrangement and reinforcement layout. All three reached strand-controlled ultimate failure. I-4 was additionally simulated with the common selected settings, but reached strand rupture instead of the observed longitudinal tensile-flange splitting. Its peak load was overpredicted by 25.4%. The symmetry constraint restricts the kinematic development of the observed splitting mechanism; without a corresponding unsymmetrical comparison, the total discrepancy is not attributed exclusively or quantitatively to symmetry. I-4 is retained as a boundary-of-validity case and excluded from the detailed local-field assessment of the strand-controlled specimens [25].

Material laws and transferability: The effective localisation length *l_ch_* = 37.5 mm, identified as (h/4) for the 150 mm deep flexural specimens, is specific to the investigated fibre–matrix system and calibration procedure. Its transfer to different fibre systems, mixtures or specimen geometries requires renewed identification; a general proportionality between localisation length and specimen depth has not been established.

Likewise, the compressive law was derived from cube tests and thus reflects a partially restrained stress state; its use for the connector region rests on the mechanical argument that comparable confinement is present there, supported by the push-out observations [23], but was not verified by dedicated triaxial testing. Since the mix design was not disclosed, transfer of the calibrated laws to nominally similar UHPFRC mixtures requires independent material characterisation.

**Bond behaviour of the strands**: The bond–slip law was derived from tests with limited embedment lengths. Its transfer to the substantially larger development length in the beam therefore required a length-scale correction based on the experimentally reported transition from bond-controlled failure to strand rupture. This correction represents an engineering approximation rather than a universal scaling law.

Engineering assumptions. The common interface parameter set was adopted by engineering judgement and was not calibrated separately for individual contacts. Other selected parameters of the fracture–plastic formulation were assigned on the basis of recommended values and engineering judgement. Since no comprehensive sensitivity study of these quantities was performed, their individual contribution to the remaining differences between numerical and experimental response cannot be quantified independently. The four-path representation of B550B reinforcement may overestimate local bond transfer through its larger summed perimeter. The magnitude of this effect was not separately quantified, and the local bond response of the conventional reinforcement is not independently validated.

Failure criterion and residual mesh dependency: Strand failure is evaluated as attainment of the ultimate strain defined in the constitutive law rather than by an explicit fracture formulation; the reported ultimate state therefore corresponds to a strain-based criterion. With Tension Characteristic Size fixed at 25 mm, the mesh study gives a 1.9% range in peak load and a 14.3% range in peak-load deflection relative to the smallest respective value. A common ultimate failure mechanism therefore does not imply mesh-independent deformation capacity. Peak-load deflection and deflection at the subsequent load drop are distinct quantities, particularly where the maximum load precedes attainment of the strand-failure criterion.

**Loading and time-dependent effects:** The analysis is restricted to monotonic, quasi-static loading. Cyclic degradation and fatigue are not represented, and time-dependent prestress losses due to creep, shrinkage and relaxation were not modelled explicitly. Fibre orientation and distribution are accounted for in a smeared sense only, whereas the scatter observed in the flexural tension tests indicates that local deviations from a homogeneous fibre arrangement occur even under laboratory conditions.

## 6. Conclusions

This study presents an experimentally based finite element modelling strategy for prestressed I-shaped UHPFRC–steel composite beams with continuous puzzle-shaped shear connectors. Building on two preceding experimental campaigns, the objective was to reproduce the global structural response and governing failure mechanisms while providing access to internal stress and strain states that could not be resolved experimentally. The main findings are summarised as follows:

- The initial constituent material relationships were established primarily from dedicated laboratory tests. The tensile and compressive UHPFRC relationships and the S460 ML steel response were derived from material testing, while the strand bond formulation was based on pull-out and anchorage investigations. Material-specimen identification was followed by structural model selection on I-2, including the compression-law alternative, the 35 mm reference mesh and the 25 mm Tension Characteristic Size. I-2 is therefore a calibration and model-selection case rather than a purely independent validation specimen.
- For the investigated UHPFRC and flexural test configuration, an effective localisation length of 37.5 mm (h/4) provided the closest overall agreement with the mean experimental flexural response among the investigated alternatives. This value was identified by inverse analysis and is specific to the tested fibre–matrix system and calibration procedure. In compression, the cube-derived relationship was retained as a confinement-informed modelling approximation after comparison with the cylinder-derived response. At the original 35 mm tensile-reference setting, only the cube-based analysis continued to the strand-controlled ultimate failure mode. This preliminary comparison is distinct from the final 25 mm tensile-reference study and does not establish a uniquely validated confinement representation. The adopted compression law should therefore be regarded as specific to the present modelling framework rather than as a general uniaxial UHPFRC relationship.
- Following selection on I-2, the 35 mm mesh and 25 mm Tension Characteristic Size were transferred without further adjustment to I-1 and I-3. Numerical peak loads of 982.6, 1396.0 and 1377.3 kN differ from the plotted experimental maxima by +10.1%, +4.9% and +0.7%, respectively. The corresponding peak-load-deflection deviations are +8.3%, +1.0% and −12.7%. The separate fixed-reference mesh study indicates relatively stable peak loads but remaining deformation sensitivity.
- The simulations reproduced the experimentally observed strand-controlled failure of beams I-1 to I-3 and the development of inter-dowel web yielding in the S460 ML steel web. Qualitative comparison with DIC contours further showed spatial correspondence in the location and spatial extent of strain localisation, including the transition from the concentrated response of I-1 to the extended localisation pattern of the four-point bending specimens I-2 and I-3. The additional I-4 analysis overpredicted the peak load by 25.4% and reached strand rupture instead of longitudinal splitting; it therefore defines a limitation of the symmetry-constrained framework rather than an additional successful failure-mode validation.
- The three-dimensional models of the strand-controlled specimens provide additional insight into the connector-level UHPFRC response and indicate the development of inter-dowel web yielding in the steel web. Local tensile splitting demand, compressive inelasticity and transverse flange deformation develop simultaneously around the continuous connectors and vary consistently with the global bending-moment distribution. These numerically interpreted internal states provide a mechanical interpretation of experimentally inferred failure mechanisms that could not be directly resolved by surface-based measurements.

Overall, the selected framework provides useful global and qualitative local agreement for the investigated strand-controlled members, while its prediction of deformation remains sensitive to the numerical reference settings and discretisation. Material-test identification, structural reference-model selection and subsequent transfer assessment must be distinguished. The unsuccessful reproduction of I-4 splitting confines the demonstrated scope of the symmetry-constrained model. The framework is not a universal UHPFRC parameter set: transfer to other material systems or governing failure mechanisms requires renewed characterisation and assessment.

## Figures and Tables

**Table 1:** UHPFRC matrix and fibre reinforcement used in all experimental campaigns.

Parameter	Value/Description
Matrix concept	Fine-grained, self-compacting UHPC matrix, optimised for particle packing density
Constituent classes	Portland cement, silica fume, quartz powder, graded quartz aggregate, water, polycarboxylate-ether superplasticiser
Cement type and content	CEM I 42.5 R; 720 kg/m^3^
Water-to-binder ratio	0.23
Maximum aggregate size	0.8 mm
Fibre type	Prototype straight ultra-high-strength steel fibres (voestalpine Special Wire GmbH)
Fibre tensile strength	≈3800 MPa (production-related scatter 3600–4000 MPa)
Fibre diameter	<120 μm
Fibre aspect ratio l/d	≈75
Fibre volume content (beam campaign)	2.0 Vol. %
Detailed mix proportions	Not disclosed (proprietary formulation of the project partner)

**Table 2:** S460 ML steel properties before and after thermal cutting. All strength values in MPa.

Test	Producer-Provided Strengths		Properties After Thermal Cutting			
	Rp0.2	Rm	Rp0.2,i	Rp0.2	Rm,i	Rm
20 mm_1	487	599	473	mean:	541	mean:
20 mm_2			470	481	529	544
20 mm_3			488		551	
20 mm_4			490	difference:	554	difference:
20 mm_5			484	1.27%	545	9.22%
20 mm_6			480		543	

**Table 3:** Input Parameters for the parameter study of the tensile stress/strain relationship.

Variants:	First Crack Strain [−] V1	Valley After Drop Strain [−] V2	Second Peak Strain [−] V3
1/4 h	0	0.0004	0.00266667
1/3 h	0	0.0003	0.002
1/2 h	0	0.0002	0.00133333
2/3 h	0	0.00015	0.001

**Table 4:** Mesh sensitivity of I-2 at a constant 25 mm Tension Characteristic Size; deflections are paired with the respective peak loads.

	I-2 Experimental	I-2 FE 50 mm	I-2 FE 35 mm	I-2 FE 25 mm
Peak load [kN]	1331.0	1422.3	1396.0	1407.1
Load deviation [%]	—	+6.9	+4.9	+5.7
Deflection at peak load [mm]	120.2	116.6	121.5	133.3
Deflection deviation [%]	—	−3.0	+1.0	+10.9

**Table 5:** Peak loads and corresponding centre deflections for the strand-controlled specimens I-1 to I-3; deviations are relative to the experimental curve.

Beam	Fmax Exp. [kN]	Fmax FE [kN]	Load Deviation [%]	Deflection at Fmax Exp. [mm]	Deflection at Fmax FE [mm]	Deflection Deviation [%]
I-1	892.6	982.6	+10.1	70.1	75.9	+8.3
I-2	1331.0	1396.0	+4.9	120.2	121.5	+1.0
I-3	1367.7	1377.3	+0.7	132.0	115.3	−12.7

## Data Availability

The data presented in this study are available on reasonable request from the corresponding author. The data are not publicly available because they are part of an ongoing doctoral research project and would require substantial additional curation for independent reuse.

## Appendix A. S460 ML Steel Input Parameters

**Basic**		
Young s Modulus-E [MPa]	200,000	
Poisson s Ratio-MU	0.3	
Tension Strength-FT [MPa]	481	
Compression Strength-FC [MPa]	−481	
**Tensile**		
Tension Function	0	0.895773294908742
	0.00153	1
	0.00999	1.01789667126091
	0.03142	1.08760389257821
	0.05954	1.12600760138663
	0.08283	1.13262749028944
	0.10722	1.11095100864553
	0.15247	0.939731863175041
	0.17247	0.751785490540033
	0.19247	0.501190327026688
Tension Characteristic Size [m]	0.035 (unchanged structural-steel reference size)	
Tension Localisation Onset	0.00153	
Fixed Crack	1.0	
**Compressive**		
Compressive Function	−0.19247	0.501190327026688
	−0.17247	0.751785490540033
	−0.15247	0.939731863175041
	−0.10722	1.11095100864553
	−0.08283	1.13262749028944
	−0.05954	1.12600760138663
	−0.03142	1.08760389257821
	−0.00999	1.01789667126091
	−0.00153	1
	0	0.895773294908742
Compressive Characteristic Size	0.035	
Compression Localisation Onset	−0.00153	
**Shear**		
Shear Stiffness Function	0.0	1.0
Shear Strength Function	0.0	1.0
Shear Localisation Onset	0.0	
Tension-Compressive		
Ft Reduction-Compred	0.0	1.0
Fc Reduction-Compred	0.0	1.0
**Miscellaneous**		
Excentricity-EXC	1	
Dir of pl Flow-BETA	0.0	
Rho-Density [kton/m^3^]	0.00785	
Thermal Expansion-Alpha [C^−1^]	0.000012	
Activate Substepping	selected	
SUBSTEPS PER FT	3	
MAX SUBSTEPS	100	
**Element Geometry**		
Geometrical Non-Linearity	Linear	
Idealisation	3D	
Non-Quadratic Element	selected	

## Appendix B. Prestressing Strands 1660/1860 Input Parameters

**EC2**		
Type of reinforcement	Tendon	
Young’s Modulus E [GPa]	197	
Characteristic Yield Strength f xk [MPa]	1760	
Epsilon u k [−]	0.04	
Parameter k [−]	1.09	
Safety Format	Mean	
Last Generation Epsilon u k	0.04	
Last Generation Parameter k	1.09	
**Basic**		
Material Prototype	CCReinforcement	
Reinf 01 Young s Modulus [GPa]	197	
Area [mm^2^]	150	
**Reinf Function**		
Reinf 01 Yield Strength YS [MPa]	1760	
Reinf 01 Number of Multilinear	7	
Reinf 01 eps2 [−]	0.01	
Reinf 01 f2 [MPa]	1760	
Reinf 01 eps3 [−]	0.012	
Reinf 01 f3 [MPa]	1815	
Reinf 01 eps4 [−]	0.015	
Reinf 01 f4 [MPa]	1860	
Reinf 01 eps5 [−]	0.02	
Reinf 01 f5 [MPa]	1895	
Reinf 01 eps6 [−]	0.03	
Reinf 01 f6 [MPa]	1915	
Reinf 01 eps7 [−]	0.04	
Reinf 01 f7 [MPa]	1920	
**Miscellaneous**		
Rho-Density [kg/m^3^]	7850	
Thermal Expansion-Alpha [C^−1^]	0.000012	
Active in Compression	selected	
**Element Geometry**		
Geometrical Non-Linearity	Nonlinear	
Geom Type	Bar with Bond	
Embedded Reinforcement [mm]	Minimum 1	
Embed Short Bars	selected	
Default Application, Application from Interval	1	
**Bar With Bond**		
Bar End	Fixed NONE	
Bar Perimeter [m]	0.04932300466135975	
Max Bond Strength [MPa]	13.15	
Function Bond Slip	0	0.302664933970246
	0.0000625	0.441386362039942
	0.000125	0.567496751194211
	0.0001875	0.599024348482779
	0.00025	0.69360714034848
	0.000375	0.756662334925615
	0.0005	0.81971752950275
	0.00075	0.93
	0.001	1.0
	0.002	1
	0.01	0.8

## Appendix C. B 550 B Input Parameters

**EC2**	
Type of reinforcement	Reinforcement
Young s Modulus [GPa]	200
Characteristic Yield Strength f xk [Mpa]	550
Class of Reinforcement	B
Safety Format	Mean
**Basic**	
Material Prototype	CCReinforcement
Reinf 01 Young’s Modulus-E [GPa]	200
Profile [mm]	4/5/7, respectively (each 1D path represents one quarter of the physical bar area)
Number of Profiles	1
Area	Automatically calculated respectively
**Reinf Function**	
Reinf 01 Yield Strength [Mpa]	605
Reinf 01 Number of Multilinear	2
Reinf 01 eps2 [−]	0.05
Reinf 01 f2 [Mpa]	653.4
**Miscellaneous**	
Rho-Density [kg/m^3^]	7850
Thermal Expansion-Alpha [$C^{-1}$]	0.000012
**Element Geometry**	
Geometrical Non-Linearity	NONLINEAR
Geom Type	Bar with Bond
Embedded Reinforcement	Minimum 1.0e-3
Embed Short Bars	selected
Default Application, Application from Interval	1

## Appendix D. Cementitious2 SHCC HPFRC Input Parameter ATENA Science

**Generate Material**		
Type of material	HPFRC	
**Basic**		
Material Prototype	CC3DNonLinCementitious2SHCC	
Young s Modulus [MPa]	44,600	
Poisson s Ratio-MU [−]	0.2	
Tension Strength-FT [MPa]	10.64	
Compression Strength-FC [MPa]	−154.7 (compression zone); −152.7 (tensile zone)	
**Tensile**		
Tensile function [−]	0	1
	2.67 × 10^−5^	0.897556390977444
	0.000106666666666668	0.771616541353384
	0.00016	0.705545112781955
	0.000186666666666668	0.691165413533835
	0.000240000000000001	0.684116541353383
	0.000400000000000001	0.684868421052632
	0.000666666666666666	0.716071428571428
	0.000799999999999999	0.732330827067669
	0.00186666666666667	0.804135338345865
	0.00266666666666667	0.845488721804511
	0.004	0.835714285714286
	0.0048	0.82406015037594
	0.00586666666666667	0.803571428571429
	0.00933333333333334	0.793233082706767
	0.0128	0.79906015037594
	0.0178666666666667	0.751503759398496
	0.0266666666666667	0.673684210526316
	0.0666666666666667	0.217387218045113
	0.112	0.0429511278195489
	0.167442666666667	0
Tension Characteristic Size [m]	0.025 (reference and fixed-reference mesh study); 0.035 and 0.050 (tensile reference-size sensitivity only)	
Tension Localisation Onset [−]	0	
Fixed crack	0	
Activate Aggregate Interlock [m]	0.0008	
**Compressive**		
Compressive Function	−0.0587128781719258	0.2
	−0.0337128781719258	0.25
	−0.0137873461701365	0.328069821175327
	−0.0110011059775993	0.381896743011223
	−0.0091158510647141	0.467746935023288
	−0.00778330140741369	0.558034455569828
	−0.00682931699366454	0.685599085287508
	−0.0045124977031309	0.865197734278796
	−0.00288466715586052	0.983084208271403
	−0.00219567841259725	0.973680716039161
	−0.00177168533981985	0.898209423176495
	−0.00094641310887812	0.68452256320285
	0	0.333109783697622
Compressive Characteristic Size	0.035 (fixed for the updated studies)	
Compression Localisation Onset	−0.00177168533981985	
**Fibre Reinforcement**		
Fibre Volume Fraction	0.02	
Fibre E Modulus	200,000	
Fibre Shear Modulus	77,000	
Fibre Cross Section Factor	0.9	
Fibre Diameter	0.000115	
**Tension-Compressive**		
Ft Reduction-COMPRED	0.0	1
	1	1
Fc Reduction-COMPRED	0	1.00
	0.6	1.00
**Miscellaneous**		
Excentricity-EXC	0.52	
Dir of pl Flow-BETA	0.0	
Rho-Density	0.002440	
Thermal Expansion-Alpha	0.000012	
FT Multiplier	2.1	
**Element Geometry**		
Geometrical Non-Linearity	Nonlinear	
Idealisation	3D	
Non-Quadratic Element	selected	

## Appendix E. Interface Parameters

The single parameter set below applies to the steel loading plate–UHPFRC, steel support plate–UHPFRC and steel web–UHPFRC interfaces, including the continuous shear connectors. These values were adopted as simplified engineering assumptions without contact-specific calibration.

**Basic**	
Material Prototype	CC3DInterface
Normal Stiffness-K NN [MN/m^3^]	5.13 × 10^7^
Tangential Stiffness-K TT [MN/m^3^]	5.13 × 10^7^
Cohesion [MPa]	1.0
Friction coefficient [−]	0.4
Tensile strength-FT [MPa]	0.3
Ellipse in tension	selected
**Miscellaneous**	
Min Norm Stiff-K NN MIN [MN/m^3^]	5.13 × 10^5^
Min Tang Stiff-K TT MIN	5.13 × 10^5^
Moving Interface	No
**Element Geometry**	
Geometrical Non-Linearity	Linear
Define Local x Direction	Automatic
